# Out-of-pocket Expenses and Time Spent on Clinic Visits Among HIV Pre-exposure Prophylaxis Users and Other Clinic Attendees in Eswatini

**DOI:** 10.1007/s10461-022-03859-3

**Published:** 2022-10-11

**Authors:** Stefan Kohler, Shona Dalal, Anita Hettema, Sindy Matse, Till Bärnighausen, Nicolas Paul

**Affiliations:** 1 Heidelberg Institute of Global Health, Medical Faculty and University Hospital, Heidelberg University, Heidelberg, Germany; 2 Institute for Social Medicine, Epidemiology and Health Economics, Charité – Universitätsmedizin Berlin, corporate member of Freie Universität Berlin and Humboldt Universität zu Berlin, Berlin, Germany; 3 Department of Global HIV, Hepatitis and Sexually Transmitted Infections Programmes, World Health Organization, Geneva, Switzerland; 4 Clinton Health Access Initiative, Mbabane, Eswatini; 5 Eswatini Ministry of Health, Mbabane, Eswatini

**Keywords:** Cost analysis, Eswatini, Health expenditures, HIV prevention and control, HIV pre-exposure prophylaxis, Opportunity costs, Out-of-pocket expenses, Transportation costs

## Abstract

User costs constitute a barrier to the uptake of HIV pre-exposure prophylaxis (PrEP), but their magnitude appears rarely assessed. In this prospective observational study, we assessed self-reported out-of-pocket expenses (OOPE) and time spent on clinic visits during a PrEP demonstration project in Eswatini. At six public primary care clinics, 240 PrEP users and other clinic attendees were interviewed after a clinic visit. Among the 79.2% of clinic attendees reporting any medical OOPE (e.g., expenses for consultations or drugs) and/or non-medical OOPE (e.g., expenses for transport, food, or phone use), the median total OOPE was $1.36 (IQR 0.91–1.96). Non-medical OOPE occurred mostly due to transport expenses. The median travel time for a clinic visit was 1.0 h (IQR 0.67–2.0). The median time spent in the clinic was 2.0 h (IQR 1.15–3.0). The median opportunity cost of a clinic visit was $7.54 (IQR 5.42–11) when valuing time spent on a clinic visit with Eswatini’s per-capita gross domestic product. Our findings can guide measures to reduce the user costs of PrEP in Eswatini and other contexts in which oral PrEP is provided through health care facilities.

**Supplementary Information:**

The online version contains supplementary material available at 10.1007/s10461-022-03859-3.

## Resumen

Los costes de los usuarios constituyen un obstáculo para la adopción de la profilaxis previa a la exposición al VIH (PrEP), pero su magnitud rara vez se evalúa. En este estudio observacional prospectivo, evaluamos los gastos de bolsillo (OOPE) declarados por los propios usuarios y el tiempo dedicado a las visitas clínicas durante un proyecto de demostración de la PrEP en Eswatini. En seis clínicas públicas de atención primaria, se entrevistó a 240 usuarios de la PrEP y a otros asistentes a la clínica después de una visita a la misma. Entre el 79,2% de los asistentes a las clínicas que declararon algún OOPE médico (por ejemplo, gastos por consultas o medicamentos) y/o OOPE no médico (por ejemplo, gastos de transporte, comida o uso del teléfono), la mediana del OOPE total fue de 1,36 dólares (IQR: 0,91–1,96). Los gastos no médicos se debieron principalmente a los gastos de transporte. La mediana del tiempo de viaje para una visita a la clínica fue de 1,0 horas (IQR 0,67–2,0). La mediana del tiempo empleado en la clínica fue de 2 horas (IQR 1,15–3,0). La mediana del coste de oportunidad de una visita a la clínica fue de 7,65 dólares (IQR 5,55–11) al valorar el tiempo dedicado a una visita a la clínica con el producto interior bruto per cápita de Eswatini. Nuestros resultados pueden orientar las medidas para reducir los costes de uso de la PrEP en Eswatini y en otros contextos en los que se suministra la PrEP oral a través de los centros de salud.

## Introduction

The addition of oral HIV pre-exposure prophylaxis (PrEP) to other HIV prevention interventions (e.g., risk-reduction counselling and HIV testing) has reduced HIV transmission by up to 86% in multiple randomized controlled trials [1–7]. The World Health Organization (WHO) recommends PrEP for individuals at substantial risk of HIV infection since 2015 [8]. PrEP is also part of WHO’s recommended public health approach to HIV prevention and treatment [9]. By the end of 2019, 120 countries had adopted PrEP recommendations in their national guidelines [10]. By early 2022, most PrEP initiations have been performed in South Africa (415,658) followed by Zambia (233,471), the US (207,047), Nigeria (223,312), Uganda (222,956), and Kenya (157,571) [11]. In Eswatini, which had an estimated HIV prevalence of 26.8% among adults aged 15 to 49 years and 4800 new HIV infections in 2020 [12], PrEP has been initiated 34,076 times since becoming available, initially through demonstration projects [11]. Since 2019, PrEP has been scaled-up nationwide and become available in more than 200 health care facilities, including government, mission, and private clinics. In all public health facilities, PrEP is provided freely. In private facilities, PrEP drugs are free of charge, but clients might pay a consultation fee.

While PrEP is effective in preventing HIV infection when taken regularly, discontinuation and inadequate adherence to PrEP are common [13, 14]. In the studied demonstration project, 35.7% of those who initiated PrEP did not return for any follow-up visit [15]. A study from a large-scale routine PrEP delivery project in Kenya found that only 31% of those who initiated PrEP returned for their one-month follow-up to a health facility [16]. PrEP user costs, such as out-of-pocket expenses (OOPE) and the time spent on accessing PrEP services, may reduce PrEP uptake and adherence and thereby impede efforts to expand PrEP. Studying OOPE for community health services, a cross sectional study of 17,944 rural and urban service users in Latin America, India, Cuba, and Nigeria found that OOPE were common and negatively correlated with health service utilization [17]. Studies of PrEP users in the US found that higher co-payments for PrEP pills reduced adherence [18], whereas monthly co-payments of $20 or less were associated with higher PrEP retention [19]. Qualitative studies with people who discontinued PrEP in Kenya [20] and Eswatini [21] described transportation costs and clinic opening hours coinciding with work schedules as reasons for PrEP discontinuation. In qualitative studies of men having sex with men in the US, co-payments and reimbursement difficulties were not only named as reasons for PrEP discontinuation [22], but co-payments were also named as reasons to reduce the willingness to initiate PrEP use [23].

Few studies appear to have assessed medical OOPE in relation to PrEP drug co-payments [18, 19, 24, 25]. Only one study assessed medical OOPE for PrEP clinic visits [25]. To our knowledge, no studies of medical OOPE, non-medical OOPE, or time spent on PrEP use have been conducted in low-income or middle-income countries. In the study at hand, we collected cost data from PrEP users and other clinic attendees during a 2017–2019 PrEP demonstration project in the Hhohho region of Eswatini. Our aims were, first, to measure the OOPE and opportunity costs related to attending PrEP demonstration project clinics in Eswatini; second, to assess if and how OOPE and opportunity costs varied between PrEP users and those attending the clinic for other health services.

## Methods

### Study Setting

Eswatini is a landlocked country with a GDP per capita of 4215 US$ in 2021 [26]. In 2017, 29% of its 1.1 million people lived in the northwestern Hhohho region [27]. Between August 1, 2017, and January 31, 2019, a PrEP demonstration project was conducted in six nurse-led public-sector primary care clinics in Hhohho. Oral PrEP was offered as an additional HIV prevention method to all clinic attendees aged 16 or older who had a substantial risk of HIV infection in a risk assessment, were interested in PrEP, HIV-negative, and had no contra-indication for tenofovir disoproxil fumarate or lamivudine (e.g., kidney disease or pancreatitis) [15, 28]. Four demonstration project clinics were in rural areas in northern Hhohho, two were in closer proximity to the capital Mbabane. The demonstration project clinics had participated in a previous trial on early antiretroviral treatment and were purposively selected by the Eswatini Ministry of Health to represent middle to high volume health facilities [15]. Like other HIV services, PrEP was provided free of charge. The demonstration project was an active research site, and a PrEP promotion package was introduced during the project [15].

### Study Design and Population

We conducted a prospective observational study throughout the PrEP demonstration project. Research assistants travelled from Mbabane to a clinic and interviewed selected clinic attendees after their clinic visit. Study days were randomly scheduled on weekdays between Monday and Thursday. A sampling strategy that reduces the risk of oversampling clinic attendees who had longer consultations was applied [29]. The final sample size was determined by pragmatic aspects such as the availability of a research assistant on a scheduled study day and the travel time to a clinic. Tablet-assisted personal interviews were conducted based on a self-developed questionnaire with new, adapted, and previously used questions [30, 31] (Supplement A). The interview included questions about different categories of OOPE, the arrival time at the clinic, travel time to the clinic, forgone earning opportunities, and lost income. Questions were pre-tested to assess if the content and duration was acceptable. Responses were recorded anonymously. Three groups of attendees were interviewed: (1) clinic attendees using only PrEP services, (2) clinic attendees using PrEP and other health services, and (3) clinic attendees using only health services other than PrEP.

### Clinic Attendance Cost Measures

Medical OOPE were estimated as the sum of expenses for consultation, medical tests, laboratory tests, non-PrEP drugs, and PrEP drugs. Non-medical OOPE were estimated as the sum of expenses for transport, childcare, food, or phone calls and text messages while travelling to or being at the clinic and other expenses. The time spent in the clinic was estimated as the difference between the self-reported arrival time at the clinic and the start time of the interview. Two-way travel time was estimated by doubling the self-reported travel time to the clinic. The time spent on the clinic visit was estimated as the sum of the two-way travel time and the time spent in the clinic. The opportunity cost of time was estimated by valuing time with a time cost. The total cost for clinic attendance was estimated as the sum of OOPE and the opportunity cost of time spent on the clinic visit.

### Data Analysis

We describe the socioeconomic characteristics of clinic attendees and the estimated clinic attendance costs using the median (IQR) for continuous variables and frequencies for categorical variables. For the time cost, a lower bound was estimated by valuing time with no cost if no lost income due to the clinic visit was reported and with the median of the lost income per time spent on the clinic visit if lost income was reported. An upper bound of the time cost was estimated by valuing time with Eswatini’s 2017–2019 average gross domestic product (GDP) per capita [26], irrespective of whether lost income due to the clinic visit was reported. To calculate the average GDP per capita and work time in Eswatini, 220 workdays per year and 8 work hours per day were assumed.

Statistical differences between groups of attendees were assessed using Pearson's χ^2^ test for categorical variables. To allow for non-normal distribution, continuous variables were compared across two groups using the Wilcoxon rank-sum test and across three groups using the Kruskal–Wallis test. Multivariable regressions were used to assess the relationships between the reason(s) for a clinic visit as independent variables and the following dependent variables: medical OOPE, non-medical OOPE, time in the clinic, two-way travel time, time spent on the clinic visit, and the cost of clinic attendance. Zero-inflated dependent variables, like the OOPE and the lost income-based cost of clinic attendance, were assessed in two-part models [32]. The first part of the two-part model included a logistic regression model for the dichotomized dependent variable (e.g., if any expenses were incurred); the second part included a generalized linear model with a Gaussian distribution and log link for the non-zero values of the dependent variable (e.g., amount spent if any expenses). Regression estimates were used to calculate linear combinations of coefficients. Standard errors were estimated using the Huber–White sandwich estimator.

Data cleaning involved assuming the arrival time at the clinic and the interview start time were interchanged in four instances and reclassifying one PrEP restart as a PrEP initiation visit. Costs were recorded in Eswatini Lilangeni (SZL) and converted to United States dollar ($) using the official average exchange rate for 2018 of $1 = SZL13.234 [33]. The significance threshold was set at P < 0.05. All analyses were carried out using Stata SE 15.1.

## Results

### Study Population

A total of 240 clinic attendees were interviewed immediately after their clinic visits; 70 (29.2%) clinic attendees visited the clinic for PrEP only, 109 (45.4%) for both PrEP and other services, and 61 (25.4%) for services other than PrEP only. Almost as many men (47.1%) as women (52.9%) attended the clinic only for PrEP. More women (83.5–83.6%) than men (16.4–16.5%) visited the clinic for both PrEP and other services and for other services only. Among the 179 (74.6%) users of PrEP, 93 (38.8%) initiated PrEP during the clinic visit and 86 (35.8%) had a PrEP follow-up consultation. People attending the clinic for PrEP were older, had fewer years of education, were more likely to be employed, and more likely to have any monthly income than participants who attended the clinic for PrEP and other services or other services only. Reasons for attending the clinic were PrEP (45.8%), outpatient services (20.8%), other HIV testing and counselling (16.3%), family planning (16.3%), ante- or postnatal care (15.0%), child welfare services (12.9%), and other reasons (2.9%). A quarter (25%) of the clinic attendees reported two or more reasons for visiting the clinic (Table 1 and Supplement B Table S1).

**Table 1:** Sociodemographic characteristics, clinic visit times, and clinic visit reasons of PrEP users and other clinic attendees

	Total	PrEP only (1)	PrEP and other services (2)	Other services only (3)^a^	P
	N = 240	N = 70	N = 109	N = 61	
Socioeconomic characteristics					
Age	29 (23–35)	34 (28–40)	28 (21–33)	26 (22–34)	< 0.001
Female	179 (74.6)	37 (52.9)	91 (83.5)	51 (83.6)	< 0.001
Education (none)	19 (7.9)	11 (15.7)	4 (3.7)	4 (6.6)	0.041
Primary education	70 (29.2)	25 (35.7)	29 (26.6)	16 (26.2)	
Secondary education	133 (55.4)	29 (41.4)	67 (61.5)	37 (60.7)	
Tertiary education	18 (7.5)	5 (7.1)	9 (8.3)	4 (6.6)	
Unemployed	144 (60.0)	32 (45.7)	69 (63.3)	43 (70.5)	0.01
Any monthly income	190 (79.2)	65 (92.9)	84 (77.1)	41 (67.2)	0.001
If any, amount ($)	298 (76–1032)	756 (189–1133)	151 (76–831)	151 (45–529)	0.002
Forgone earning opportunity^b^	41 (17.1)	13 (18.6)	19 (17.4)	9 (14.8)	0.84
Any lost income^b^	23 (9.6)	5 (7.1)	12 (11.0)	6 (9.8)	0.69
If any, amount ($)	4.53 (3.78–11)	7.56 (3.78–11)	4.53 (2.04–9.45)	5.67 (3.78–11)	0.70
Clinic visit time					
6 am to 8 am	60 (25.0)	19 (27.1)	34 (31.2)	7 (11.5)	0.044
9 am to 10 am	113 (47.1)	30 (42.9)	51 (46.8)	32 (52.5)	
11 am to 2 pm	67 (27.9)	21 (30.0)	24 (22.0)	22 (36.1)	
Clinic visit reasons^c^					
PrEP initiation	24 (10.0)	12 (17.1)	12 (11.0)	0 (0)	0.004
PrEP follow-up	86 (35.8)	58 (82.9)	28 (25.7)	0 (0)	< 0.001
Outpatient department	50 (20.8)	0 (0)	31 (28.4)	19 (31.1)	< 0.001
HIV testing and counseling	39 (16.3)	0 (0)	25 (22.9)	14 (23.0)	< 0.001
Family planning	39 (16.3)	0 (0)	26 (23.9)	13 (21.3)	< 0.001
Ante- or postnatal care	36 (15.0)	0 (0)	22 (20.2)	14 (23.0)	< 0.001
Child welfare	31 (12.9)	0 (0)	21 (19.3)	10 (16.4)	< 0.001
Other^d^	7 (2.9)	0 (0)	5 (4.6)	2 (3.3)	0.20

n (%) or median (interquartile range). Groups were compared using Pearson's χ2 and Kruskal–Wallis tests. P-values for pairwise comparisons, a comparison of PrEP use (1 + 2) vs. other services use (3), and a comparison of PrEP or other service use (1 + 3) vs. PrEP and other services use (2) are provided in Supplement B Table S1

^a^Including three persons who were only counselled about PrEP

^b^Due to clinic visit

^c^Multiple reasons possible

^d^Including two persons accompanying another clinic attendee, two persons collecting antiretroviral therapy for a partner, one person asking to continue PrEP at the clinic, one person attending the clinic for cancer screening, and one person helping at the clinic

### Out-of-pocket Expenses for Clinic Visits

Most (79.2%) clinic attendees reported OOPE, 20.8% reported no OOPE. If any OOPE were reported, the median amount was $1.36 (IQR 0.91–1.96). Medical OOPE were reported by 31.7% of clinic attendees. If any medical OOPE were reported, the median amount was $0.38 (IQR 0.38–0.76). Non-medical OOPE were reported by more clinic attendees (73.3%; Pearson's χ^2^ P < 0.001). If any non-medical OOPE were reported, the median amount was $1.21 (IQR 0.91–1.81), which was substantially higher than the median amount of medical OOPE (Wilcoxon rank-sum test P < 0.001). For medical OOPE, 30.4% of the clinic attendees reported consultation fees (median $0.38 [IQR 0.38–0.76]), one participant (0.4%) reported spending $0.38 for medical tests, and 2.9% reported expenses for non-PrEP drugs (median $0.60 [IQR 0.38–0.83]). No clinic attendee reported expenses for PrEP drugs. Non-medical OOPE included expenses for transport, food, phone use, and childcare. Transport costs were reported by 65.8% of the clinic attendees (median $1.06 [IQR 0.91–1.51] for a roundtrip from their home to the clinic). Expenses on food were reported by 23.3% of the interviewed clinic attendees, expenses on phone calls or text messages by 15.8%, and childcare expenses by 1.7%. No other expenses in relation to the clinic visit were reported (Table 2 and Supplement B Table S2).

**Table 2:** Out-of-pocket expenses, time spent on clinic visits, and total cost of clinic attendance among PrEP users and other clinic attendees

	Total	PrEP only (1)	PrEP and other services (2)	Other services only (3)	P
	N = 240	N = 70	N = 109	N = 61	
Out-of-pocket expenses					
Any OOPE	190 (79.2)	46 (65.7)	92 (84.4)	52 (85.2)	0.004
If any, amount ($)	1.36 (0.91–1.96)	1.40 (0.91–2.45)	1.28 (0.91–1.89)	1.51 (0.94–1.93)	0.55
Medical out-of-pocket expenses					
Any medical OOPE	76 (31.7)	5 (7.1)	44 (40.4)	27 (44.3)	< 0.001
If any, amount ($)	0.38 (0.38–0.76)	0.38 (0.38–0.38)	0.38 (0.38–0.38)	0.38 (0.38–1.06)	0.17
Any consultation expenses	73 (30.4)	5 (7.1)	44 (40.4)	24 (39.3)	< 0.001
If any, amount ($)	0.38 (0.38–0.76)	0.38 (0.38–0.38)	0.38 (0.38–0.38)	0.38 (0.38–1.06)	0.041
Any medical test expenses	1 (0.4)	0 (0)	0 (0)	1 (1.6)	0.23
If any, amount ($)	0.38 (0.38–0.38)			0.38 (0.38–0.38)	
Any non-PrEP drug expenses	7 (2.9)	0 (0)	3 (2.8)	4 (6.6)	0.083
If any, amount ($)	0.60 (0.38–0.83)		0.83 (0.60–0.83)	0.38 (0.26–0.76)	0.28
Any PrEP drug expenses	0 (0)	0 (0)	0 (0)	0 (0)	
Non-medical out-of-pocket expenses					
Any non-medical OOPE	176 (73.3)	46 (65.7)	84 (77.1)	46 (75.4)	0.22
If any, amount ($)	1.21 (0.91–1.81)	1.17 (0.91–2.27)	1.06 (0.91–1.66)	1.28 (0.91–1.66)	0.24
Any transport expenses	158 (65.8)	44 (62.9)	74 (67.9)	40 (65.6)	0.79
If any, amount (two-way, $)	1.06 (0.91–1.51)	1.13 (0.91–2.27)	1.06 (0.91–1.51)	1.06 (0.91–1.51)	0.16
Any food expenses	56 (23.3)	9 (12.9)	26 (23.9)	21 (34.4)	0.014
If any, amount ($)	0.42 (0.30–0.76)	0.38 (0.38–0.49)	0.60 (0.30–0.83)	0.38 (0.30–0.76)	0.34
Any phone expenses	38 (15.8)	8 (11.4)	18 (16.5)	12 (19.7)	0.42
If any, amount ($)	0.38 (0.15–0.45)	0.34 (0.15–0.60)	0.38 (0.20–0.45)	0.30 (0.15–0.42)	0.72
Any childcare expenses	4 (1.7)	0 (0)	2 (1.8)	2 (3.3)	0.34
If any, amount ($)	3.78 (2.27–5.67)		5.67 (3.78–7.56)	2.27 (0.76–3.78)	0.22
Other out-of-pocket expenses					
Any other expenses	0 (0)	0 (0)	0 (0)	0 (0)	
Total out-of-pocket expenses					
Medical OOPE ($)	0 (0–0.38)	0 (0–0)	0 (0–0.38)	0 (0–0.38)	< 0.001
Non-medical OOPE ($)	0.91 (0–1.51)	0.91 (0–1.66)	0.91 (0.20–1.51)	1.13 (0.15–1.51)	0.77
All OOPE ($)	1.06 (0.38–1.81)	0.91 (0–1.66)	1.10 (0.57–1.81)	1.28 (0.76–1.81)	0.18
Time spent on clinic visit					
Time spent on clinic visit (h)	3.34 (2.40–4.75)	3.09 (2.22–4.72)	3.77 (2.73–4.98)	2.92 (2.27–3.83)	0.014
Time in clinic (h)	1.98 (1.15–2.99)	1.88 (0.90–2.55)	2.63 (1.47–3.27)	1.58 (1.00–2.38)	< 0.001
PrEP initiation (h)	2.13 (1.28–3.13)	2.52 (1.37–2.62)	2.65 (1.58–3.46)	1.58 (1.00–2.38)	< 0.001
PrEP follow-up (h)	1.68 (0.90–2.68)	1.68 (0.90–2.37)	2.53 (0.82–2.90)	1.58 (1.00–2.38)	0.30
Two-way travel time (h)	1.00 (0.67–2.00)	1.17 (0.83–2.00)	1.00 (0.67–2.00)	1.00 (0.67–2.00)	0.40
Opportunity cost of time spent on clinic visit					
Time cost (lost income, $)^a^	0 (0–0)	0 (0–0)	0 (0–0)	0 (0–0)	0.68
Time cost (GDP, $)^b^	7.54 (5.42–11)	6.98 (5.00–11)	8.50 (6.17–11)	6.58 (5.12–8.65)	0.014
Total cost of clinic attendance					
Cost of clinic visit (lost income, $)^a^	1.21 (0.38–2.27)	0.91 (0–2.27)	1.28 (0.76–2.27)	1.36 (0.91–1.96)	0.100
Cost of clinic visit (GDP, $)^b^	8.92 (6.51–12)	8.34 (6.21–12)	10 (7.15–13)	8.02 (6.36–11)	0.054

n (%) or median (interquartile range). GDP = gross domestic product. OOPE = out-of-pocket expenses. Groups were compared using Pearson's χ2 and Kruskal–Wallis tests. P-values for pairwise comparisons, a comparison of PrEP use (1 + 2) vs. other services use (3), and a comparison of PrEP or other service use (1 + 3) vs. PrEP and other services use (2) are provided in Supplement B Table S2

^a^Time valued with the median of ¢3.03 per minute lost income of those who reported lost income

^b^Everyone’s time valued with a per-capita GDP of ¢3.76 per minute worktime

**Fig. 1:**
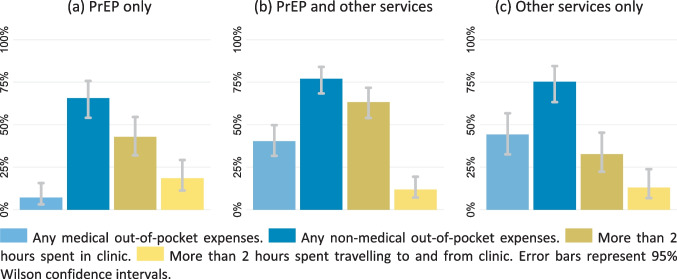
Out-of-pocket expenses and time spent on clinic visits among PrEP users and other clinic attendees

People attending the clinic for PrEP only were less likely to have any OOPE (65.7%) than those attending the clinic for both PrEP and other services (84.4%; Pearson's χ^2^ P = 0.004) or other services only (85.2%; Pearson's χ^2^ P = 0.01). The lower likelihood of OOPE among PrEP-only users was driven by less frequent medical OOPE (7.1%) compared to other clinic attendees (41.8%; Pearson's χ^2^ P < 0.001). Comparing the total OOPE across all clinic attendees, users of only PrEP had lower medical OOPE (median $0 [IQR 0–0]) than users of PrEP and other services (median $0 [IQR 0–0.38]; Wilcoxon rank-sum test P < 0.001) and users of other services only (median $0 [IQR 0–0.38]; Wilcoxon rank-sum test P < 0.001). Total non-medical OOPE (Kruskal–Wallis test P = 0.77) and total OOPE (Kruskal–Wallis test P = 0.18) were similar among all groups of clinic attendees (Table 2 and Fig. 1).

### Time Spent on Clinic Visits

The median time that clinic attendees spent in the clinic was 2.0 h (IQR 1.15–3.0). The median travel time for a roundtrip to the clinic was 1.0 h (IQR 0.67–2.0). Both added-up to a median time of 3.3 h (IQR 2.4–4.75) spent on a clinic visit. People attending the clinic for PrEP only (median 1.9 h [IQR 0.9–2.55]) or other services only (median 1.6 h [IQR 1.0–2.4]) spent less time in the clinic than clients who used PrEP and other services (median 2.6 h [IQR 1.5–3.3]; both Wilcoxon rank-sum tests P < 0.001). Across the health services used, the median time spent in the clinic was 58–163% higher than the time spent on travelling to the clinic and back (Table 2 and Fig. 1).

### Opportunity Cost and Total Cost of Clinic Attendance

About one in six (17.1%) clinic attendees reported a forgone opportunity to earn money due to the clinic visit. Fewer clinic attendees (9.6%) reported that they actually lost income due to the clinic visit. The proportion of clinic attendees forgoing an earning opportunity or income as well as the amount of income lost due to the clinic visit was not statistically different between users of PrEP only, users of both PrEP and other services, and users of other services only. Among the clinic attendees who reported lost income, the median income lost was $4.53 (IQR 3.78–11). The median time cost of those reporting lost income due to the clinic visit was ¢3.03 (IQR 0.98–7.27) per minute (Table 1).

Using the median lost income of ¢3.03 per minute to value the time of clinic attendees who reported lost income and a time cost of zero for other clinic attendees, we estimated no opportunity cost related to the clinic visits for most clinic attendees (median $0 [IQR 0–0]). In contrast, when valuing the time of all clinic attendees with the GDP per capita and minute worktime (¢3.76), we estimated a median opportunity cost of $7.54 (IQR 5.42–11) per clinic visit. As clinic attendees who used both PrEP and other health services spent more time at the clinic, they had a higher median opportunity cost than other clinic attendees when valuing time with the per-capita GDP ($8.50 [IQR 6.17–11] vs. $6.77 [IQR 5.08–10]; Wilcoxon rank-sum test P = 0.005) (Table 2).

### Clinic Visit Reasons and Costs

Multivariable regressions of the medical and non-medical OOPE on the reasons for a clinic visit indicate that visiting the outpatient department and antenatal or postnatal care were more often associated with medical OOPE than using other health services. Visiting the outpatient department and attending a clinic for other reasons than PrEP, the outpatient department, HIV services, familiy planning, ante- or postnatal care, and child welfare was associated with additional expenses when medical OOPE occurred. Neither the odds of having non-medical OOPE nor travel time were associated with any clinic visit reason (Table 3).

**Table 3:** Regression analyses of the relationships of out-of-pocket expenses, time spent on clinic visit, and total cost of clinic attendance with clinic visit reasons

Covariates (model)	(1.1) Any medical OOPE^a^ *OR (95% CI)*	(2.1) Any non-medical OOPE^a^ *OR (95% CI)*	(3) Time in clinic (minutes) *Coef. (95% CI)*	(5) Time spent on clinic visit (minutes) *Coef. (95% CI)*	(7.1) Any costs of clinic attendance^a,c^ *OR (95% CI)*
Clinic visit reason					
PrEP initiation	0.96 (0.45 to 2.05)	1.15 (0.54 to 2.47)	55 (32 to 77)***	53 (24 to 83)***	1.17 (0.46 to 2.97)
PrEP follow-up	0.60 (0.22 to 1.62)	0.70 (0.29 to 1.68)	21 (− 4.66 to 46)	26 (− 7.08 to 59)	0.61 (0.21 to 1.73)
Outpatient department	33 (11 to 96)***	0.93 (0.40 to 2.17)	− 0.19 (− 27 to 27)	− 17 (− 52 to 19)	2.89 (0.87 to 9.64)
HIV testing and counseling	0.94 (0.33 to 2.67)	0.61 (0.28 to 1.35)	6.65 (− 18 to 32)	15 (− 20 to 50)	0.76 (0.3 to 1.89)
Family planning	2.49 (0.87 to 7.08)	1.26 (0.5 to 3.19)	6.51 (− 19 to 32)	− 7.29 (− 41 to 27)	1.87 (0.65 to 5.36)
Ante- or postnatal care	6.06 (2.12 to 17)***	1.61 (0.61 to 4.25)	57 (28 to 86)***	64 (28 to 100)***	2.82 (0.86 to 9.22)
Child welfare	2.86 (0.93 to 8.75)	2.25 (0.8 to 6.33)	15 (− 11 to 40)	2.20 (− 31 to 35)	1.77 (0.55 to 5.70)
Other	0.53 (0.12 to 2.28)	2.58 (0.27 to 24)	25 (− 24 to 74)	43 (− 36 to 122)	1.95 (0.21 to 19)
Constant	0.12 (0.04 to 0.35)***	2.67 (1.12 to 6.39)*	86 (61 to 111)***	179 (145 to 213)***	2.97 (1.02 to 8.65)*
Pseudo or adjusted R^2^	0.29	0.030	0.12	0.070	0.069

Covariates (model)	(1.2) If any medical OOPE, amount [ln($)]^a^ *Coef. (95% CI)*	(2.2) If any non-medical OOPE, amount [ln($)]^a^ *Coef. (95% CI)*	(4) Two-way travel time (minutes) *Coef. (95% CI)*	(6) Cost of clinic attendance ($)^b^ *Coef. (95% CI)*	(7.2) If any costs of clinic attendance, amount [ln($)]^a,c^ *Coef. (95% CI)*
Clinic visit reason					
PrEP initiation	− 0.16 (− 0.49 to 0.16)	0.055 (− 0.20 to 0.31)	− 1.72 (− 24 to 20)	2.12 (0.81 to 3.43)**	0.080 (− 0.30 to 0.46)
PrEP follow-up	− 0.19 (− 0.66 to 0.28)	0.030 (− 0.25 to 0.31)	5.23 (− 19 to 30)	0.82 (− 0.59 to 2.24)	− 0.14 (− 0.56 to 0.28)
Outpatient department	0.40 (0.020 to 0.78)*	− 0.18 (− 0.47 to 0.12)	− 16 (− 42 to 9.25)	− 0.41 (− 2.00 to 1.18)	− 0.098 (− 0.47 to 0.28)
HIV testing and counseling	− 0.24 (− 0.54 to 0.056)	0.33 (− 0.04 to 0.71)	8.06 (− 20 to 36)	0.80 (− 0.86 to 2.45)	0.27 (− 0.13 to 0.68)
Family planning	− 0.035 (− 0.47 to 0.40)	− 0.094 (− 0.44 to 0.25)	− 14 (− 41 to 13)	− 0.33 (− 1.87 to 1.21)	− 0.29 (− 0.64 to 0.061)
Ante- or postnatal care	− 0.033 (− 0.49 to 0.42)	− 0.29 (− 0.59 to 0.022)	6.99 (− 22 to 36)	2.31 (0.78 to 3.85)**	− 0.27 (− 0.77 to 0.23)
Child welfare	0.22 (− 0.24 to 0.68)	− 0.29 (− 0.59 to 0.0031)	− 12 (− 38 to 13)	− 0.012 (− 1.47 to 1.45)	− 0.015 (− 0.46 to 0.43)
Other	0.75 (0.46 to 1.04)***	− 0.12 (− 0.69 to 0.44)	18 (− 28 to 65)	1.77 (− 1.74 to 5.28)	− 0.51 (− 1.14 to 0.11)
Constant	− 0.69 (− 1.17 to − 0.20)**	0.54 (0.24 to 0.83)***	94 (68 to 119)***	8.08 (6.57 to 9.59)***	1.01 (0.59 to 1.43)***
AIC/BIC or adjusted R^2^	1.02/ − 276	3.05/ − 786	− 0.0075	0.050	3.91/ − 807

^a^Two-part regression model combining a logistic regression model and a generalized linear model with a Gaussian distribution and log link

^b^Based on time valuation with per-capita gross domestic product

^c^Based on time valuation with median lost income for those who reported lost income

﻿*P < 0.05, **P < 0.01, ***P < 0.001. Additional regression analyses are provided in Supplement B Tables S3–S5

Controlling for other clinic visit reasons, we estimated that clinic attendees spent on average 141 min (95% CI 118 to 164) in the clinic for PrEP initiation and 106 min (95% CI 90 to 123) for PrEP follow-up. Travelling to the clinic and back home added, on average, 92 min (95% CI 67 to 116) to PrEP initiation and 99 min (95% CI 80 to 117) to PrEP follow-up visits. Using the GDP-based time valuation, a clinic visit for PrEP initiation was associated with additional cost of $2.12 (95% CI 0.81 to 3.43) for those already at the clinic for other services and a total cost of $10 (95% CI 8.69 to 12) for those attending the clinic for PrEP initiation only. PrEP follow-up was not associated with additional cost for those already at the clinic and had a total cost of $8.90 (95% CI 7.81 to 10) for those attending the clinic for PrEP follow-up only when using the GDP-based time valuation.

At the clinic, PrEP initiation required on average 35 min (95% CI 2.33 to 68) longer than other health services and 32 min (95% CI 6.06 to 59) longer than PrEP follow-up. PrEP follow-up required about the same time as other health services (P = 0.82). The PrEP promotion package, which was introduced stepwise during the demonstration project, was neither associated with medical OOPE, nor non-medical OOPE, nor the time spent on the clinic visit (Supplement B Tables S3–S5).

## Discussion

### Summary of Findings

Our results identified transportation and time spent on clinic visits as main causes of user costs in a PrEP demonstration project in Eswatini that provided oral PrEP through public primary care clinics to everyone at substantial risk of HIV infection. Almost eight in ten clinic attendees incurred OOPE, with a median OOPE of $1.36 (IQR 0.91–1.96). People attending the clinic only for PrEP reported medical OOPE less often than those seeking other health services. Consultation fees were the single medical OOPE incurred by some PrEP users. Non-medical OOPE occurred more often and were substantially higher than medical OOPE, but neither the frequency nor the amount of the non-medical OOPE were associated with the health services used at the clinic. Irrespective of encountering financial expenses in relation to a clinic visit, clinic visits took time. Interviewed clinic attendees spent a median time of 1.0 h (IQR 0.67–2.0) travelling to the clinic and back home and a median time of 2.0 h (IQR 1.15–3.0) in the clinic. This time cannot or only partly be used for other purposes and thus poses an opportunity cost to clinic attendees. While travel time did not vary significantly with the services used at the clinic, participants spent more time in the clinic if they used PrEP together with other services. Multivariable regression analyses showed that PrEP initiation tended to require more time at the clinic than PrEP follow-up or the use of other health services than PrEP.

### Value of Time Spent on Clinic Visits

To compare time to other costs, we valued the time of clinic attendees in monetary terms. We derived a monetary value of time based on either the self-reported income lost due to the clinic visit or GDP per capita. The estimated value of a time unit was ¢3.03 per minute when derived from lost income and ¢3.76 per minute when derived from GDP per capita. Further, a monetary value was only assigned to time in the lost income-based valuation if any lost income was reported. As only one in ten clinic attendees reported lost income due to the clinic visit, the median time cost for a clinic visit was zero when using the lost income-based time valuation. When valuing all time equally with the GDP per capita and minute, the median time cost of clinic attendees increased to $7.54 (IQR 5.42–11). Both approaches to value time have been used before in economic evaluations [34, 35]. The GDP-derived time value can be interpreted as an average societal value of time in Eswatini, whereas the lost income-derived time value is more person specific.

In the lost income-based valuation, we estimated the opportunity cost of lost paid worktime but not the opportunity cost of lost leisure time, lost opportunities to earn money, and lost unpaid worktime (e.g., household work, care work, volunteer work, or subsistence farming) [34, 36]. We interpreted the income-based valuation as a lower bound of the value of time. This lower bound could reflect the narrow time cost of a PrEP clinic visit from the perspective of employees and employers. The GDP-based valuation uniformly assigned a higher time cost to every minute spent on a clinic visit. We interpreted the GDP-based opportunity cost as an upper bound of the time value, which could reflect the broader cost of a PrEP clinic visit from a societal perspective (compare, e.g., [37])﻿. The opportunity cost range defined by the lower and upper bounds illustrates that time costs might vary from negligible, when considering only lost income, to exceeding OOPE by far, when considering lost paid and unpaid time.

### Comparison with Previous Findings

In previous studies, costs of PrEP have been assessed predominantly from a provider or health system perspective, for instance, in South Africa [38–42], Brazil [43], Ukraine, Peru [38], Nigeria [44], Uganda [45], South Korea [46], and India [47]. Few previous studies assessed OOPE in relation to PrEP. One study, which assessed self-reported OOPE in a PrEP demonstration project in five community health centers in the US, found that 54.2% of PrEP users incurred OOPE with a quarterly median of $34 [25]. About two thirds of OOPE were incurred due to PrEP medication and one third due to clinic visit costs [25]. Another analysis among PrEP users in the US revealed monthly PrEP medication co-payments of a similar magnitude, with an average of $34 (SD 66) [18]. Two other studies from the US retrospectively assessed drug payments and co-payments for PrEP using prescription data. The first study estimated OOPE of $54–94 for a monthly supply of PrEP [25]. The second study estimated that 77% of the PrEP users had monthly co-payments of $20 or less, with average co-payments of $20 (SD 78) [19].

Our study complements previous findings by documenting PrEP-related medical and non-medical OOPE in a low-income setting and opportunity costs of time, which have been rarely quantified before. A cost-effectiveness analysis of PrEP in Canada assumed, without providing a justification, that four hours of work were missed per outpatient PrEP visit [48]. This assumed time cost is similar in magnitude to our estimates. The study then used age group-stratified average hourly wages, age group-stratified employment rates, and the age distribution among workers in Canada to calculate opportunity costs of 2012 Can$67.93 per 4 h clinic visit [48]. In comparison, we estimated an opportunity cost of $0–7.54 per clinic visit. The magnitude of the opportunity cost of time for a PrEP-related clinic visit appears similar across settings (1.3‰ and 1.9‰ of the GDP per capita) after considering wealth differences between Canada (GDP per capita $52,669 in 2012) and Eswatini (average GDP per capita $3972 in 2017–2019) [26].

In our study, people who attended a clinic only for PrEP services spent a median of 3.09 h on their clinic visit; 7.1% of them reported medical OOPE and 65.7% non-medical OOPE, mostly transportation costs. Two qualitative studies from Kenya [20] and the same demonstration project in Eswatini [21] among people who discontinued PrEP identified transportation costs and clinic opening hours coinciding with work as reasons for PrEP service discontinuation. For medical OOPE, qualitative [22] and quantitative studies [18, 19] among PrEP users in the US identified co-payments and gaps in cost coverage as reasons for low adherence and discontinuation.

### Practical Implications

Non-medical OOPE and time costs frequently pose barriers to PrEP use. Reducing user costs could help reduce the risk of underutilizing HIV prevention related to a potentially low willingness to pay for prevention [23, 49]. Subsidizing transport expenses, lowering the distance to a PrEP provider, and/or reducing the time required in a clinic for PrEP might offer ways to address these known barriers. In a qualitative study that was conducted during the same demonstration project in Eswatini, PrEP users, health care workers, and policymakers recommended reaching out to the communities and providing PrEP outside the hospital environment to increase uptake [50]. Others have similarly argued that community PrEP dispensing outside of HIV treatment clinics is a key facilitator of PrEP scale-up [51]. In the context of the COVID-19 pandemic, the WHO recommended multi-months pill refills, telehealth follow-up, and community-based PrEP dispensing [52]. All these measures should reduce the user costs of seeking PrEP care. As some might be easier to implement (e.g., transport subsidies or more widely spaced pill refills) than others (e.g., shorter clinic processes, telehealth follow-up, or community-based PrEP), a combination of these measures could help to reduce PrEP user costs in the short and long run.

The findings this study, together with further data, also allow for a crude assessment of how PrEP users spent time at a clinic. In a complementary study of the human resource needs for PrEP provision in the same demonstration project, we estimated that health care workers spent, on average, 29 min on PrEP initiation and 16 min on PrEP follow-up [53]. The discrepancy of the time spent by health care workers on PrEP provision and the estimated time spent by clients in the clinic for PrEP initiation (141 min) and follow-up (106 min) indicates potential for reducing waiting time during PrEP clinic visits. Notwithstanding the user costs of accessing PrEP through a health facility, another study from the same demonstration projects elicited that PrEP users had different preferences about the PrEP uptake and delivery setting at the clinic [54]. Tailoring PrEP care to user preferences could be complementary to reducing user costs in efforts to increase PrEP use.

### PrEP Care After the Demonstration Project

This study has been conducted in six primary care clinics of a PrEP demonstration project in Eswatini, which preceded a nationwide PrEP scale-up. In 2021, 42 clinics in Hhohho provided PrEP according to our information. The PrEP scale-up should have decreased commuting distances to a clinic, but transportation costs have reportedly increased in the past years. Therefore, we continue to expect transportation costs to pose a barrier to clinic-based PrEP utilization. PrEP-related tests and drugs remained free of charge after the demonstration project, but PrEP initiation and follow-up have presumably become shorter as the frequency of creatinine testing has been reduced during the nationwide scale-up. We previously estimated that drawing blood for a creatinine test takes 5 min [55]. Reducing the duration of a PrEP clinic visit by 5 min has a relatively small effect on the estimated time that PrEP users spend in the clinic for PrEP (106–141 min) and on the total time spent on a  PrEP clinic visit (205–233 min). Hence, we also expect that time costs continue to be a barrier to clinic-based PrEP utilization.

### Strengths and Limitations

Strengths of our study include, first, a collection of data on different categories of OOPE as well as time spent on clinic visits. Second, data were collected from randomly chosen attendees immediately after their clinic visit. Third, study days were prospectively selected at random. The study is also subject to limitations. First, a relatively small number of clinic attendees was sampled. Hence, the conducted analyses might have been underpowered to detect small effects, and changes over time were not investigated. Second, data were self-reported and included recalled expenses. Third, the study clinics were not randomly selected, and research assistants might not have been able to select clients from all consultation rooms at a clinic. Fourth, the preset schedule of study days was sometimes adjusted. Fifth, research assistants might have missed clinic attendees that arrived in the early morning, especially at the clinics furthest away from Mbabane. The three latter limitations could have introduced a selection bias to the conducted interviews. This concern is mitigated by the presumption that expenses and commuting times may be roughly similar across workdays, visit times, and clinics offering PrEP. Finally, PrEP users were more likely than other users to have participated in an interview more than once. While this could reflect actual clinic utilization, the anonymous data collection prevented us from accounting for repeated sampling in the statistical analysis.

## Conclusion

OOPE were common among the attendees of six public primary care clinics of a PrEP demonstration project in the Hhohho region of Eswatini. In contrast to the clients attending a clinic for other services than PrEP, the clients attending a clinic only for PrEP rarely incurred medical OOPE and never reported expenses for PrEP pills or medical tests. Transport expenses were the most common non-medical OOPE for all clinic attendees. Notwithstanding financial expenses, clinic attendees spent a substantial amount of time in the clinic, especially when initiating PrEP, and on travelling to and from a clinic. GDP per capita-based time valuation illustrated how opportunity costs of time can substantially exceed the financial expenses for a clinic visit. Providing transport subsidies, more widely spaced pill refills, shorter clinic processes, telehealth follow-up, or community-based PrEP could be measures that increase PrEP use and adherence by reducing non-medical OOPE and the time spent on accessing PrEP.

## Supplementary Information

Below is the link to the electronic supplementary material.

Supplementary file1 (PDF 3070 KB)

Supplementary file2 (DOCX 123 KB)

## Data Availability

The data and code that support the findings of this study are openly available in heiDATA at 10.11588/data/OYUCUZ.
