# Supplementary material for: Out-of-pocket Expenses and Time Spent on Clinic Visits Among HIV Pre-exposure Prophylaxis Users and Other Clinic Attendees in Eswatini
Source: AIDS Behav. 2022 Oct 11;27(4):1222–33. doi: 10.1007/s10461-022-03859-3 (PMC9551250; doi:10.1007/s10461-022-03859-3)
Supplement: Supplementary file 1 — Supplementary file1 (PDF 3070 KB) [file 10461_2022_3859_MOESM1_ESM.pdf]

PATIENT EXIT INTERVIEW
PrEP

Date: |__|__| |__|__| |__|__|__|__|
Day Month Year

Name of health facility: ____

Name of interviewer: ____

Verbal consent

This clinic is participating in a demonstration project on Pre-exposure prophylaxis, a new HIV prevention strategy. We would like to ask you a few questions about your clinic visit today and use the findings to inform the Ministry of Health about clients experiences and knowledge about PrEP as well as and the cost that clients get to access PrEP services. Your responses will be anonymous, and you do not need to give us your name. Do you agree with us recording your replies and using your data for analysis?

Lomtfolampilo ungenele lucwaningo lwe Pre-exposure prophylaxis, indlela lensha yekuvikela I HIV. Ngingatsandza kukubuta imibuta lemibalwa mayelana neluvakasho lwakho lwasemtfolampilo namuhla bese sisebentisa imiphumela kwatisa litiko letempilo mayelana nemivo nelwati labanalo nge PrEP kanye netindleko. Timphendvulo takho titoba yimfihlo futsi akudzingeki kutsi usiphe libito lakho. Uyavuma kutsi sicophe timphendvulo takho nekutsi sisebentise iminingwano yakho kuze sihlatiye?

Verbal consent provided for PrEP exit interview/ Imvume ngemlomo yekuyekela locociswano lwe PrEP:
☐ Yes Yebo
☐ No Cha

Time at start of interview: |__|__| : |__|__|
Sikhatsi sekucala kucocisana:
Hours (0-24) Minutes (0-60)

Supplement A

1.	Basic identifying & sociodemographic information	
1.1	*What is the sex of the respondent?* **(only ask if not obvious)** Bulili	☐ Female Sifazane<br>☐ Male Silisa<br>☐ Transgender XX
1.2	How old are you? *Please enter the age in years.* Mingakhi iminyaka yakho?	|__|__| years Iminyaka<br>**If age less than 16, stop the questionnaire here.**
1.3	Are you in a relationship? Ingabe kukhona lovana naye? *Please tick what applies.*	☐ Single Cha<br>☐ Partner, not living together Ukhona mane asihlali kanyekanye<br>☐ Partner, living together Ukhona, sihlala kanyekanye<br>☐ Multiple partner Banengi
1.4	What is the highest level of school you have attended? Wafundza kangananі eskolweni? *Tick what applies.*	☐ None Kute<br>☐ Primary school Esikolweni lesincane<br>☐ Secondary Esikolweni lesikhulu<br>☐ Tertiary Sikolwa sekucecesha
1.5	Do you currently work to earn money or obtain food? Uyasebenta yini nyalo kuze utfole imali nobe kudla?<br>Tick all that apply. Multiple answers are possible.	☐ Working full-time for a salary Ngiyasebenta ngicashiwe<br>☐ Working part-time for a salary Ngihle ngisebenta hhayi sonkhe sikhatsi<br>☐ Selling goods Ngitsengisa tintfo<br>☐ Subsistence farming including animal rearing Siyalima phindze sifuye<br>☐ Unemployed Angisebenti<br>☐ Other, specify Lokunye, kusho: _____________
1.6	What type of clinic visit did you attend today? Utele luphi lusito lapha emtfolamphilo namuhla?<br><br>Tick all that apply. Multiple answers are possible.	☐ HIV testing and counseling Kuhlola nekwelulekwa nge HIV<br>☐ Out patient department Kutolashwa phindze ngijike<br>☐ Antenatal care Kupopola sisu<br>☐ Post natal care Kunakekelwa kwalababelekile<br>☐ Famiy planning Kuhlela umndeni<br>☐ PrEP initiation visit Ngitocala I PrEP<br>☐ PrEP follow up visit Ngitolandzelelwa ku PrEP<br>☐ Other Lokunye:______________________<br><br>If client came for ART refill or ART initiation, **stop the questionnaire.**
1.7	How satisfied are you with the services you recieved today? Wenetiseke kangananі nelusito lolutfolile namuhla?	☐ Very satisfied Ngenetiseke kakhulu<br>☐ Somewhat satisfied Ngenetisekile nje<br>☐ Not satisfied Angikanetiseki<br>☐ Very unsatisfied Angikanetiseki sanhlobo
1.8	Have you been interviewed before about your experiences on PrEP? Ukile yini wabutwa imibuto mayelana ngemivo yakho nge PrEP?	☐ Yes Yebo [If yes, continue with Q1.9)<br>☐ No Cha [If no, continue with Q2.1)
1.9	How many times have you been interviewed before? Ubutwe kangakhi lemibuto phambilini?	☐ _________

2.	**PrEP knowledge and experience**	
**2.1**	Where did you first hear about PrEP? Weva kuphi nge PrEP kwekucala ngca?<br><br>Allow client to answer first and then probe with responses for missing topics, if needed. Tick all that apply. Multiple answers are possible.	☐ The health education session in this facility Uma sifundziswa ngetemphilo lamtfolampilo<br>☐ Individual counselling by HCW Kwelulekwa sisebenti setempilo uwedvwa<br>☐ Educational video in the clinic Lifilimu lelifundzisako kumabonakudze walamtfolampilo<br>☐ Poster in the waiting room in the clinic Liphepha lekufundzisa lelinamatsiselwe ebondzeni emtfolamphilo<br>☐ Flyer/ brochure given to me in the clinic Lipheshana labanginika lona emtfolamphilo<br>☐ Peer referral card Likhadi lekundlulisela embili umlingani wakho<br>☐ Friends Bangani<br>☐ Family Tihlobo<br>☐ Television Kumabonakudze<br>☐ Newspaper Ephepheni<br>☐ Radio Emsakatweni<br>☐ Internet XX<br>☐ Community worker Kuloosebentela emmangweni<br>☐ Community event Umcimbi wasemmangweni:<br>______________________<br>☐ I have not heard of PrEP before Angikake ngeva nge PrEP ngaphambilini.<br>☐ Other Lokunye: _________________________
**2.2**	Are you starting PrEP today or did you receive a PrEP refill today? Uyayitsatsa yini iPrEP nyalo?	☐ Yes Yebo [If yes, continue with Q2.4)<br>☐ No Cha [If no, continue with Q2.3)
**2.3**	Have you previously been on PrEP? Ukile wayitsatsa yini iPrEP ngaphambilini?	☐ Yes Yebo [If yes, continue with Q2.4)<br>☐ No Cha [If no, continue with Q2.22)
**2.4**	Did you receive counselling before starting PrEP? Ululekiwe yini ngaphambi kwekutsi utsatse iPrEP?	☐ Yes Yebo<br>☐ No Cha [If no, continue with Q2.6)
**2.5**	Which topics were discussed during the counselling session? Kwakhulunywa ngani ngesikhatsi bakweluleka?<br><br>Allow client to answer first and then probe with responses for missing topics, if needed. Tick all that apply. Multiple answers are possible.	☐ Taking a pill a day consistently Kutsatsa liphilisi linye onkhe malanga<br>☐ Benefits of PrEP Lokuhle kwe PrEP<br>☐ Limitations of PrEP Lokubi kwe PrEP<br>☐ Possible side effects Imivuka lengahle ibe khona<br>☐ Need for good adherence Sidzingo sekunatsa emaphilisi ngendlela lefanele<br>☐ 3-monthly HIV testing Kuhlolwa kwesimo sengati njalo emva kwetinyanga letitsatfu<br>☐ Need for consistent condom use Sidzingo sekusebentisa lijazi lemkhwenyana njalonjalo<br>☐ Follow up appointments Kuyolandzelelwa<br>☐ Other Lokunye: _____________________<br>☐ I don't remember Angkhumbuli.

**2.6**	For how long have you been taking PrEP? Sewuyitsatse sikhatsi lesinganani iPrEP?	☐ Started PrEP today Ngicale namuhla<br>☐ 1 month Inyanga yinye<br>☐ 2-3 months Tinyanga letimbili kuya kuletintsatfu<br>☐ 4-6 months Tinyanga letine kuya kuletisitfupha<br>☐ 7-9 month Tinyanga letisikhombisa kuya letiyimfica<br>☐ 10-12 month Tinyanga letilishumi kuya kuletilishumi nakubili<br>☐ More than 12 months Kwengca etinyangeni letilishumi nakubili<br>☐ I can't remember Angikhumbuli
**2.7**	In the last 30 days how would you rate your ability to take PrEP as prescribed? Emalangeni langu30 lengcile,ingabe ungatsi ukhone kanganani kutsatsa iPrEP ngendlela lefanele? [if client was initiated today on PrEP, continue with Q2.22]	☐ Very good (I take PrEP every day or almost every day) Kahle kakhulu (ngitsatsa iPrEP cishe onkhe Malanga)<br>☐ Fair (I take PrEP 3-4 times week) Ngiyayinatsa nje (ngitsatsa I PrEP emalanga lamatsatfu kuya kulamane ngeliviki)<br>☐ Poor (I only take PrEP once or twice a week) Angiyinatsi kahle (ngitsatsa I PrEP Kanye noma kabili ngeliviki)<br>☐ I did not take PrEP at all Angiyitsatsi sanhlobo iPrEP
**2.8**	During today's visit, did you receive counselling about PrEP adherence? Eluvakashweni lwakho lwanamuhla, ingabe bakwelulekile yini ngemigomo yekunatsa iPrEP?	☐ Yes Yebo<br>☐ No Cha
**2.9**	What do you think will happen if you don't take PrEP daily, as prescribed? Ngekucabanga kwakho,kwentekalani uma ungayitsatsi iPrEP ngendlela lefanele?<br><br>Allow client to answer first and then probe with responses, if needed. Tick all that apply. Multiple answers are possible.	☐ I don't know Angati<br>☐ I am at a higher risk of getting HIV Ngiba sezingeni lelisetulu lekutfola ligciwane le HIV<br>☐ I will get sick Ngitogula<br>☐ Nothing will happen Kute lokutokwenteka<br>☐ Other Lokunye: ___________________________
**2.10**	What are some reasons that make it difficult to take PrEP as prescribed? (once a day at the same time) Yini sizatfu lesibangela kutsi kube nebulukhuni kulandzela imitsetfo yekutsatsa iPrEP?<br><br>Allow client to answer first and then probe with responses, if needed. Tick all that apply. Multiple answers are possible.	☐ Side effects Imivuka<br>☐ Not remembering because of busy or inconsistent schedule Ngihlale ngimatasatasa ngako ngiyakhohlwa<br>☐ Alcohol use Kunatsa tjwala<br>☐ Unsupportive partner(s) Lengitsandzana naye akanginaki<br>☐ I have had no trouble taking PrEP as prescribed Ngite inkinga yekutsatsa iPrEP ngendlela lefanele<br>☐ Other Lokunye: ________________________
**2.11**	Since you started taking PrEP, have you had any side-effects that you attribute to taking PrEP? Solo wacala kutsatsa iPrEP, ingabe ikhona yini imivuka lokubi lehambelana nekutsatsa iPrEP?	☐ Yes Yebo<br>☐ No Cha [if 'no' continue with Q 2.13]

**2.12**	[If the answer to 2.9 is YES] What side-effects have you had Yini lemivuka lobe nayo<br><br>Allow client to answer first and then probe with responses, if needed. Tick all that apply. Multiple answers are possible.	☐ Mild gastrointestinal (nausea, bloating, gas) Kungaphatseki kahle esiswini (kunyakuluka, kucumba,kusuta)<br>☐ Vomiting Kuhlanta<br>☐ Diarrhoea Umsheko<br>☐ Weight loss Ngiyehla emtimbeni<br>☐ Weight gain Ngiyakhuluphala emtimbeni<br>☐ Headache Ngiphatfwa yinhloko<br>☐ Other Lokunye: ________________________
**2.13**	Since you started taking PrEP, have you told any of your friends, partners or family about your PrEP use? Solo wacala kutsatsa iPrEP, kukhona yini bangani, tingani noma lilunga lemndeni wakho lolutjelile ngayo?	☐ Yes Yebo<br>☐ No Cha [If no, continue with Q 2.15]
**2.14**	Who have you told about your PrEP use? Check all that apply. Utjele bani kutsi uyayisebentisa I PrEP?	☐ Main partner Singani<br>☐ Casual partner Makhwapheni<br>☐ Family member Lilunga lemndeni<br>☐ Friends Bangani<br>☐ Other, please specify:____________________
**2.15**	What are some reasons you have not told people about your PrEP use? Titsini tizatfu tekutsi ungatjeli bantfu mayelana nekutsatsa iPrEP?<br><br>Allow client to answer first and then probe with responses, if needed. Tick all that apply. Multiple answers are possible.	☐ I worry about my partner judging me. Ngikhatsatwa kutsi singani sami sitangihlulela.<br>☐ I worry about my family judging me. Ngikhatsatwa kutsi umndeni wami utangihlulela.<br>☐ I worry about my friends judging me. Ngikhatsatekile ngekutsi umndeni wami utangehlulela.<br>☐ I don't think my partner would understand my motivation for taking PrEP. Angicabangi kutsi singani sami sitocondza kutsi ngiyifunelani I PrEP.<br>☐ My main partner does not know that I have multiple partners. Singani sami asati kutsi nginaletinye tingani.<br>☐ I do not want my partner to know that I do not trust him/her Angifuni singani sami sati kutsi angisetsembi.<br>☐ Other Lokunye: ________________________<br>☐ Not applicable – I have told everyone
**2.16**	[If "main partner" is checked for 2.14] You indicated that you told your main partner about your PrEP use. How did he/ she react when you first told him/ her? Uvete kutsi lolovana naye umtjelile ngekutsatsa iPrEP. Ingabe kwamphatsa njani uma umtjela kwekucala?	☐ He/she was supportive. Ube nguloyijabulelako.<br>☐ He/she was not supportive. Akakhombis.i kunginaka.<br>☐ Neutral Ubesemkhatsini.<br>☐ Other Lokunye: ____________________
**2.17**	[If "casual partner" is checked for 2.6] You indicated that you told your casual partner about your PrEP use. How did he/ she react when you first told him/ her? Uvete kutsi makwapheni wakho umtjelile ngekutsatsa iPrEP. Ingabe kwamphatsa njani uma umtjela kwekucala?	☐ He/ she was supportive. Ube nguloyijabulelako.<br>☐ He/ she was not supportive. Akakhombisi kunginaka.<br>☐ Neutral Ubesemkhatsini.<br>☐ Other Lokunye: ____________________
**2.18**	[If "family member" is checked for 2.6] You indicated that you told a family member about your PrEP use. How did he/ she react when you first told him/ her? Uvete kutsi utjele	☐ He/ she was supportive. Ube nguloyijabulelako.<br>☐ He/ she was unsupportive. Akakhombisi kunginaka.<br>☐ Neutral. Ubesemkhatsini.

	lilunga lemndeni mayelana nekusebentisa iPrEP. Ingabe kwamphatsa njani uma umtjela kwekucala?	☐ Other Lokunye: ___________________
**2.19**	[If "friends" is checked for 2.6]<br>You indicated that you told friends about your PrEP use. How did they react when you first told them? Uvete kutsi utjelae bangani ngekusebentisa iPrEP. Ingabe kwamphatsa njani uma umtjela kwekucala?	☐ They were supportive Babe ngulabayijabulelako<br>☐ They were not supportive Abakakhombisi kunginaka<br>☐ Neutral Babesemkhatsini<br>☐ Other Lokunye: ___________________
**2.20**	Did you receive a new PrEP refill today? Ngabe utfolile yini emaphilisi akho e-PrEP bowuwalandzile namuhla?	☐ Yes [go to section 2.21]<br>☐ No [go to next question]
**2.21**	If not, what are the main reasons you are not continuing with PrEP? Nangabe cha, ngitiphi tizatfu letikubangele kutsi ungachubeki ne PrEP<br><br>Allow client to answer first and then probe with responses. Tick all that apply. Multiple answers are possible.	☐ My HIV test was Positive Luhlolo lukhombe kutsi senginalo ligciwane le HIV<br>☐ I am no longer at risk Angisekho engotini Clarify:_________________<br>☐ I have too many side-effects Nginemivuka leminengi<br>☐ I am worried about stigma Ngikhatsatwa kutsi bayangicwaya<br>☐ I do not have time to come for the clinic follow up visits Ngite sikhatsi sekuya emtfolamphilo bayongilandzelela<br>☐ I am not able to take a tablet every day Angikhoni kunatsa liphilisi onkhe malanga<br>☐ Other Lokunye: ________________________
**2.22**	Currently PrEP is only available in a tablet form, taking 1 tablet a day to reduce the risk of HIV infection. Scientists are working to make a different kind of HIV prevention medicine that would involve getting an injection in the muscle every 2 months. If in the future an injectable PrEP would become available, would you have a preference for either oral or injectable PrEP?<br><br>Kwanyalo PrEP simtfola ngelinatsa liphilisi linye onkhe Malanga kwehlisa ematfuba ekutfola iHIV. Bacwaningi basahlela kwakha tinhlobo temitsi letehlukene yekuvikela iHIV letifaka ekhatsi kujova emva kwetinyanga letimbili. Nakungenteka kube nemjovo wePrEP, unganconota liphilisi noma umjovo wePrEP?	☐ I would prefer injectable PrEP every 2 months<br>☐ I would prefer oral PrEP taken daily<br>☐ I would have no preference for either injectable or oral PrEP<br>☐ I am not interested in either injectable PrEP or daily oral PrEP

**3. Sexual behaviour**		
**3.1.**	At what age did you first start having sex? Wacala unangakhi iminyaka kulala?	☐ Age Umnyaka:____________
**3.2.**	When was the last time you had unprotected (condom-less) sex? Kunini lapho wagcina kulala khona ngaphandle kwe condom?	☐ < 1 week ago<br>☐ 1-2 weeks ago<br>☐ 3-4 weeks ago<br>☐ > 4 weeks ago<br>☐ Never<br>☐ I don't remember

**3.3.**	Out of the last 10 sex acts, how many do did you use a condom? Emahlandleni lang10 lengcile ulala, ulisebentise kangakhi lijazi lemkhwenyana?	☐ Number Inombolo: ________<br>☐ N/A
**3.4.**	Do you practice anal sex? Kuyenteka yini usebentise imbobo yangemuva mawulala?	☐ Yes Yebo<br>☐ No Cha
**3.5.**	Do you have a primary or main sexual partner? Ngabe unaye yini lovana naye lokunguyena yena (noma ngabe anikatsatsani)	☐ Yes Yebo<br>☐ No Cha
**3.6.**	[If 3.5 =”Yes”] On average, how often do you use a condom with this partner for vaginal or anal sex? Uvame kuyisebentisa kangakhi I condonm nalololala naye embotjeni yangembili noma yangemuva?	☐ Never Angiyisebentisi<br>☐ Seldom Ngiyisebentisa ngazo<br>☐ Sometimes Ngihle ngiyisebentisa<br>☐ Often Ngiyavama kuyisebentisa<br>☐ Always Sonkhe sikhatsi
**3.7.**	Do you know your primary partners HIV status? Ngabe uyasati yini simo se-HIV salona lovana naye?	☐ Yes Yebo<br>☐ No Cha
**3.8.**	[If 3.7 ‘Yes’] What is your primary partners HIV status? Sitsini lesimo sengati?	☐ HIV negative Ute ligciwane<br>☐ HIV positive Unalo ligciwane
**3.9.**	In total, with how many people have you had sexual intercourse with in the last 12 months? Masebaphelele, bangakhi bantfu lolele nabo etinyangeni leng12 letengcile?	☐ Number:___________
**3.10.**	Of these [3.9 #] partners, how many did you <u>have unprotected (condom-less) sex with?</u> Kulaba labangu [3.9 #] , bangakhi lolele nabo usebentisa i condom?	☐ Number:Inombolo: __________
**3.11.**	[Only if client is on PrEP, if client not on PrEP go to Q 3.14 or if client is HIV-positive, go to 4.1] Since starting PrEP, how has your number of partners in a one-month time period changed? Solo wacala iPrEP, lishintje kanjani lizinga lebantfu lolele nabo enyangeni yinye?<br><br>Allow client to answer first and then probe with responses, if needed.	☐ I have more partners than I did before. Senginentingani letinengi kunakucala.<br>☐ I have the same number of partners as I did before taking PrEP. Solo ngitsandzana nalabo bengitsandzana nabo.<br>☐ I have fewer partners than I did before starting PrEP. Setinciphile tingani tami lebenginato ngingakacali I PrEP.
**3.12.**	[If 3.5 = “Yes”] Since starting PrEP, how has your condom use with primary partner changed? Solo wacala iPrEP,ingabe lishintje kanjani lizinga lekusebetisa i condom nalona lovana naye?<br><br>Allow client to answer first and then probe with responses, if needed.	☐ I have decreased how often I use condoms with my primary partner Kwehlile kusebentisa kwami I condom nalebengivana naye<br>☐ My condom use with my primary partner stays the same. Iyafana indlela lesisebentisa ngayo i condom nalebengivana naye.<br>☐ I have increased how often I use condoms with my primary partner Seyikhulile indlela lesisebentisa ngayo i condom nalebengivana naye<br>☐ I do not have a primary partner Kute lengivana naye
**3.13.**	Since starting PrEP, how has your condom use with any TEMPORARY/CASUAL partners changed? Kusukela ucale i-PrEP, ngabe kushintjile yini kusebentisa kwakho i condom namakhwapheni wakho?	☐ I have decreased how often I use condoms with my temporary/ casual partners Kwehlile kusebentisa kwami i condom namakhwapheni<br>☐ My condom use with my temporary/ casual partners stays the same. Solo iyafana indlela lesisebentisa ngayo i condom namakhwapheni

	Allow client to answer first and then probe with responses, if needed.	☐ I have increased how often I use condoms with my temporary/ casual partners Seyikhulile indlela lesisebentisa ngayo i condom namakhwapheni<br>☐ I do not have a temporary/ casual parner. Ngite makhwapheni
**3.14.**	On a scale of 1-5, how likely do you think it is that you will contract HIV infection with your current lifestyle? Ku 1 kuya ku 5, ngabe utibona usematfubeni langanani kutsi ungatseleleka ngeligciwane le HIV ngalendlela lophila ngayo?	☐ 1: Very unlikely<br>☐ 2: Unlikely<br>☐ 3: Somewhat likely<br>☐ 4: Likely<br>☐ 5: Very likely
**3.15.**	What do you think you can do to reduce your risk? Ngabe ucabanga kutsi yini longakwenta kunciphisa bungoti bakho?<br><br>Allow client to answer first and then probe with responses. Tick all that apply. Multiple answers are possible.	☐ Have consistent condom use<br>☐ Take my PrEP tablets daily<br>☐ Reduce the number of my sexual partners<br>☐ Knowing my partners HIV status<br>☐ Other: ____________________

**4. HIV/AIDS knowledge**		
**4.1.**	For every 10 members of your community, how many of them do you think are currently living with HIV? Kubantfu labalishumi emmangweni wakini,bangakhi locabanga kutsi baphila neligciwane le HIV?	☐ Number:_______
**4.2.**	Can people reduce their chance of getting HIV virus by having just one uninfected sexual partner who has no other sex partners? Bantfu bangalehlisa yini lizinga lekutfola ligciwane le HIV ngekutsi babe namunye umuntfu labalala naye naye longalali nalomunye umuntfu?	☐ Yes Yebo<br>☐ No Cha<br>☐ Don't know Angati
**4.3.**	Is it possible for a healthy-looking person to have the HIV virus? Kungentaka yini kutsi umuntfu lobukeka angulophilie abeneligciwane le HIV?	☐ Yes Yebo<br>☐ No Cha<br>☐ Don't know Angati
**4.4.**	If a member of your family got infected wih the HIV virus, would you want it to remain a secret or not? Uma lilunga lemndeni wakho lingatfola ligciwane le HIV, ungafuna kutsi kube yimfihlo yini loko?	☐ Yes Yebo<br>☐ No Cha<br>☐ Don't know Angati
**4.5.**	If a member of your family become sick with HIV, would you be willing to share a house with him/her? Uma lilunga lemndeni wakho lingagula ngenca yeligciwane leHIV, ungavuma yini kumnakekela ekhaya lakho?	☐ Yes Yebo<br>☐ No Cha<br>☐ Don't know Angati
**4.6.**	In your opinion, if a female teacher has HIV but is not sick, should she be allowed to continue teaching in the school? Ngekubuka kwakho,uma thishela lomsikati aneligciwane le HIV kepha angaguli, kumele avumeleke kutsi achukeke afundzise?	☐ Yes Yebo<br>☐ No Cha<br>☐ Don't know Angati
HIV can be transmitted when an HIV-positive person has sex with a HIV-negative person; however, not every sex act leads to HIV infection<br>Ligciwane le HIV lingatseleIwana nangabe umuntfu loneligciwane le HIV alalana nemuntfu lote ligciwane; koja,		

akusiko konkhe kulalana lokubangela kutsi bantfu batseleleke ngeligciwane le HIV. Nyalo sitakucela uzame kubekisa ematfuba ekutsi kulalana lokutsite kubanga kutselelwana nge HIV.		
**4.7.**	Who is more likely to get infected with HIV, an HIV negative woman having unprotected vaginal sex with an HIV positive man or an HIV negative woman having anal sex with an HIV positive man? Ngubani losematfubeni lamakhulu ekutseleleka nge HIV, make lote ligciwane lolala na babe lonalo ngaphandle kwe condom noma make lote ligciwane lolalana na babe lonalo embotjeni yangemuva?	☐ Vaginal sex<br>☐ Anal sex<br>☐ Same risk
**4.8.**	Out of 10 of your friends, how many do you think believe they will get HIV in their lifetime? Kubangani bakho labangu 10, bangakhi locabanga kutsi bakholwa kutsi batayitfola I HIV emphilweni yabo?	☐ Number: _______________
**Now I want you to imagine someone in your community who is just like you (same age, sex, etc). Nyalo ngifuna ucabange umuntfu nje losemmangweni lofana nawe (lolingana naye ngemnyaka,ngebulili)**		
**4.9.**	How many years (in total) do you expect this individual to live if he or she is HIV-negative and remains HIV-negative the rest of his or her life? Ubheke lomuntfu lona kutsi aphile iminyaka lemingakhi nangabe ete ligciwane le HIV futsi achubeke abe bete imphilo yakhe yonkhe?	☐ Years:_________
**4.10.**	How many years (in total) do you expect this individual to live if he or she is HIV-positive, and was infected in the last month? Ubheke lomuntfu lona kutsi aphile iminyaka lemingakhi nangabe aneligciwane le HIV futsi alitfole kulenyanga lephelile?	☐ Years:_________

**5. Health service expenditure** Tindleko tekutfola lusito lwasemtfolampilo		
**5.1.**	I would now like to ask you a few questions about the costs you experience to access healthcare. This question is only about the cost for YOUR OWN healthcare, NOT for other people in your household. Ngingatsandza kukubuta imbuto lembalwa mayelana netindleko lotikhokhile kute utfole lusito emtfolamphilo.Lombuto ubuta ngetakho tindleko tetemphilo, hhayi bantfu lohlala nabo.<br><br>For your clinic visit TODAY, what type of expenses did you pay for and the amount? **Ensure that the respondent only counts expenses since he/she started travelling to the clinic until the time of the interview.** Kulokuta kwakho emtfolamphilo namuhla, hlobo luni lwetindleko lotibhadalele nekutsi tingumalini? **Ciniseka kutsi lophendvulako ubala tindleko latitfole nakacala luhambo lwakhe loluta emtfolamphilo kute kube ngulapho aphendvula lemibuto.** *Please tick the correct options and enter the amounts in Lilangeni.*	
**5.2.**	Consultation fee Imali yekubonwa ngu dokotela nobe nesi	☐ Yes yebo → How much did you pay? Malini? E|__|__|__|__| ☐ No cha
**5.3.**	Medical tests Tinhlolo letehlukahlukene	☐ Yes yebo → How much did you pay? Malini? E|__|__|__|__| ☐ No cha
**5.4.**	Medicines (not PrEP related) Imitsi (hhayi lehambelana ne PrEP)	☐ Yes yebo → How much did you pay? Malini? E|__|__|__|__| ☐ No cha
**5.5.**	Medicines (PrEP related, including cost of medication to treat side-effects of PrEP) Imitsi (lehambelana ne PrEP, Kanye netindleko temitsi yekulapha imivuka lebangwe yi PrEP)	☐ Yes yebo → How much did you pay? Malini? E|__|__|__|__| ☐ No cha

**5.6.**	Transport to get to the clinic (one way) Kugibela nawuya emntfolamphilo (kuya kuphela)	☐ Yes yebo → How much did you pay? Malini? E|__|__|__|__| ☐ No cha
**5.7.**	Payment for someone to look after your children while you are gone to the clinic. Kubhadala umuntfu losale wakubonela umtfwana uma uta lapaha emtfolamphilo	☐ Yes yebo → How much did you pay? Malini? E|__|__|__|__| ☐ No cha
**5.8.**	Food since you started travelling to the clinic until the time of the interview Kudla lokutsenge kusukela usuka ekhaya kute kube nyalo uphendvula lemibuto	☐ Yes yebo → How much did you pay? Malini? E|__|__|__|__| ☐ No cha
**5.9.**	Phone calls/SMS since you started travelling to the clinic until the time of the interview Lucingo lolushaye endleleni kute kube nyalo uphendvula lemibuto	☐ Yes yebo → How much did you pay? Malini? E|__|__|__|__| ☐ No cha
**5.10.**	Others, **specify** Lokunye, chaza:<br>____	How much did you pay? Malini?<br>E |__|__|__|__|
**5.11.**	At what time did you arrive at the clinic today? Ufike ngabani lapha emtfolamphilo namuhla? *Please enter the time. Bhala sikhatsi lapha.*<br>|__|__| : |__|__| Hours Minutes<br>(0-24)<br>Ama awa mizuzu	
**5.12.**	How much time did it take you today to get to the clinic? Utsetse sikhatsi lesingakanani kufika lapha emtfolamphilo namuhla? *Please enter the time.*<br>|__|__| : |__|__| Hours Minutes	
**5.13.**	Would you have been earning money if you were not at the clinic today? Ngabe bewutabe utfola imali tsite yini kube bewungaketi lapha emtfolamphilo namuhla?<br>☐ Yes<br>☐ No	
**5.14.**	If you would have been earning money, did you lose income from the time you took from your job to come here today? Uma ngabe bewutawutfola imali, ulahlekelwe ngumalini njengoba bewungekho emsebentini ulapha namuhla?<br>☐ Yes<br>☐ No	
**5.15.**	If you lost money, how much money did you lose? *Please enter the amount in Lilangeni. Nangabe ikhon imali lekulahlekele,* ngumalini ?<br>E__________________	
**5.16**	How much was your average monthly income over the past 12 months? Ibe ngumalini imali loyitfolile kuletinyanga letilishumi nambili letendlile *Please enter the amount in Lilangeni.*<br>E__________________	

	**How many times did you take this survey: Sowuyente kangakhi le survey?**	☐ Once<br>☐ Twice<br>☐ More than 2 times
	**Time at end of interview:**	**|__|__| : |__|__|**<br>**Hours Minutes**<br>**(0-24)**

Do you have any comments or feedback for us? Kukhona yini longakubuta? Thank you very much for your effort and time! Siyabonga sikhatsi nekubayincenye yalolucociswano!
