# Supplementary material for: Out-of-pocket Expenses and Time Spent on Clinic Visits Among HIV Pre-exposure Prophylaxis Users and Other Clinic Attendees in Eswatini
Source: AIDS Behav. 2022 Oct 11;27(4):1222–33. doi: 10.1007/s10461-022-03859-3 (PMC9551250; doi:10.1007/s10461-022-03859-3)
Supplement: Supplementary file 2 — Supplementary file2 (DOCX 123 KB) [file 10461_2022_3859_MOESM2_ESM.docx]

**Table S1:** Sociodemographic characteristics, clinic visit times, and clinic visit reasons of PrEP users and other clinic attendees

|  | **Total** | **PrEP only (1)** | **PrEP and other services (2)** | **Other services only (3)^a^** | **(1+3)** | **(2+3)** | **(1+2)** | **P** | **(1) vs. (2)** | **(1) vs. (3)** | **(2) vs. (3)** | **(1+3) vs. (2)** | **(1) vs. (2+3)** | **(1+2) vs. (3)** |
| --- | --- | --- | --- | --- | --- | --- | --- | --- | --- | --- | --- | --- | --- | --- |
|  | **N=240** | **N=70** | **N=109** | **N=61** | **N=131** | **N=170** | **N=179** |  |  |  |  |  |  |  |
| **Socioeconomic characteristics** | | | | | | | | | | | | | | |
| Age | 29 (23–35) | 34 (28–40) | 28 (21–33) | 26 (22–34) | 30 (25-37) | 27.50 (22-34) | 30 (24-36) | <0.001 | <0.001 | <0.001 | 0.67 | <0.001 | <0.001 | 0.085 |
| Age (16–20 years) | 24 (10.0) | 0 (0) | 18 (16.5) | 6 (9.8) | 6 (4.6) | 24 (14.1) | 18 (10.1) | <0.001 | <0.001 | <0.001 | 0.44 | 0.006 | <0.001 | 0.17 |
| 21–25 years | 59 (24.6) | 7 (10.0) | 30 (27.5) | 22 (36.1) | 29 (22.1) | 52 (30.6) | 37 (20.7) |  |  |  |  |  |  |  |
| 26–30 years | 55 (22.9) | 20 (28.6) | 24 (22.0) | 11 (18.0) | 31 (23.7) | 35 (20.6) | 44 (24.6) |  |  |  |  |  |  |  |
| 31–35 years | 43 (17.9) | 16 (22.9) | 19 (17.4) | 8 (13.1) | 24 (18.3) | 27 (15.9) | 35 (19.6) |  |  |  |  |  |  |  |
| 35–65 years | 59 (24.6) | 27 (38.6) | 18 (16.5) | 14 (23.0) | 41 (31.3) | 32 (18.8) | 45 (25.1) |  |  |  |  |  |  |  |
| Sex (female) | 179 (74.6) | 37 (52.9) | 91 (83.5) | 51 (83.6) | 88 (67.2) | 142 (83.5) | 128 (71.5) | <0.001 | <0.001 | <0.001 | 0.98 | 0.004 | <0.001 | 0.061 |
| Male | 61 (25.4) | 33 (47.1) | 18 (16.5) | 10 (16.4) | 43 (32.8) | 28 (16.5) | 51 (28.5) |  |  |  |  |  |  |  |
| Education (none) | 19 (7.9) | 11 (15.7) | 4 (3.7) | 4 (6.6) | 15 (11.5) | 8 (4.7) | 15 (8.4) | 0.041 | 0.008 | 0.13 | 0.84 | 0.091 | 0.006 | 0.82 |
| Primary education | 70 (29.2) | 25 (35.7) | 29 (26.6) | 16 (26.2) | 41 (31.3) | 45 (26.5) | 54 (30.2) |  |  |  |  |  |  |  |
| Secondary education | 133 (55.4) | 29 (41.4) | 67 (61.5) | 37 (60.7) | 66 (50.4) | 104 (61.2) | 96 (53.6) |  |  |  |  |  |  |  |
| Tertiary education | 18 (7.5) | 5 (7.1) | 9 (8.3) | 4 (6.6) | 9 (6.9) | 13 (7.6) | 14 (7.8) |  |  |  |  |  |  |  |
| Employment (working full-time) | 47 (19.6) | 23 (32.9) | 17 (15.6) | 7 (11.5) | 30 (22.9) | 24 (14.1) | 40 (22.3) | 0.021 | 0.025 | 0.037 | 0.50 | 0.078 | 0.009 | 0.28 |
| Working part-time | 12 (5.0) | 4 (5.7) | 5 (4.6) | 3 (4.9) | 7 (5.3) | 8 (4.7) | 9 (5.0) |  |  |  |  |  |  |  |
| Selling goods | 12 (5.0) | 4 (5.7) | 5 (4.6) | 3 (4.9) | 7 (5.3) | 8 (4.7) | 9 (5.0) |  |  |  |  |  |  |  |
| Subsistence farming | 19 (7.9) | 7 (10.0) | 7 (6.4) | 5 (8.2) | 12 (9.2) | 12 (7.1) | 14 (7.8) |  |  |  |  |  |  |  |
| Other employment | 6 (2.5) | 0 (0) | 6 (5.5) | 0 (0) | 0 (0) | 6 (3.5) | 6 (3.4) |  |  |  |  |  |  |  |
| Unemployed | 144 (60.0) | 32 (45.7) | 69 (63.3) | 43 (70.5) | 75 (57.3) | 112 (65.9) | 101 (56.4) |  |  |  |  |  |  |  |
| Monthly income ($) | 151 (28-756) | 491 (76-1133) | 91 (26-529) | 45 (0-295) | 189 (30–907) | 76 (0–453) | 151 (53–907) | <0.001 | <0.001 | <0.001 | 0.088 | 0.15 | <0.001 | <0.001 |
| Any monthly income | 190 (79.2) | 65 (92.9) | 84 (77.1) | 41 (67.2) | 106 (80.9) | 125 (73.5) | 149 (83.2) | 0.001 | 0.006 | <0.001 | 0.16 | 0.46 | <0.001 | 0.008 |
| If any, amount | 298 (76-1032) | 756 (189-1133) | 151 (76-831) | 151 (45-529) | 378 (76–1133) | 151 (76–605) | 378 (756–1133) | 0.002 | 0.009 | 0.001 | 0.31 | 0.19 | <0.001 | 0.025 |
| Monthly income (none) | 50 (20.8) | 5 (7.1) | 25 (22.9) | 20 (32.8) | 25 (19.1) | 45 (26.5) | 30 (16.8) | <0.001 | <0.001 | <0.001 | 0.17 | 0.16 | <0.001 | 0.004 |
| $1–150 | 68 (28.3) | 14 (20.0) | 37 (33.9) | 17 (27.9) | 31 (23.7) | 54 (31.8) | 51 (28.5) |  |  |  |  |  |  |  |
| $151–750 | 57 (23.8) | 17 (24.3) | 23 (21.1) | 17 (27.9) | 34 (26.0) | 40 (23.5) | 40 (22.3) |  |  |  |  |  |  |  |
| $751+ | 65 (27.1) | 34 (48.6) | 24 (22.0) | 7 (11.5) | 41 (31.3) | 31 (18.2) | 58 (32.4) |  |  |  |  |  |  |  |
| Forgone earning opportunity^b^ | 41 (17.1) | 13 (18.6) | 19 (17.4) | 9 (14.8) | 22 (16.8) | 28 (16.5) | 32 (17.9) | 0.84 | 0.85 | 0.56 | 0.65 | 0.90 | 0.69 | 0.58 |
| Lost income^b^ | 23 (9.6) | 5 (7.1) | 12 (11.0) | 6 (9.8) | 11 (8.4) | 18 (10.6) | 17 (9.5) | 0.69 | 0.39 | 0.58 | 0.81 | 0.49 | 0.41 | 0.94 |
| Lost income ($)^b^ | 0 (0–0) | 0 (0–0) | 0 (0–0) | 0 (0–0) | 0 (0–0) | 0 (0–0) | 0 (0–0) | 0.72 | 0.42 | 0.60 | 0.82 | 0.52 | 0.44 | 0.94 |
| Any lost income^b^ | 23 (9.6) | 5 (7.1) | 12 (11.0) | 6 (9.8) | 11 (8.4) | 18 (10.6) | 17 (9.5) | 0.69 | 0.39 | 0.58 | 0.81 | 0.49 | 0.41 | 0.94 |
| If any, amount | 4.53 (3.78–11) | 7.56 (3.78–11) | 4.53 (2.04–9.45) | 5.67 (3.78–11) | 7.56 (3.78–11.33) | 4.53 (2.27–11) | 4.53 (3.78–11) | 0.70 | 0.40 | 0.57 | 0.89 | 0.56 | 0.41 | 0.92 |
| **Clinic visit time** | | | | | | | | | | | | | | |
| 6 am to 8 am) | 60 (25.0) | 19 (27.1) | 34 (31.2) | 7 (11.5) | 26 (19.8) | 41 (24.1) | 53 (29.6) | 0.044 | 0.48 | 0.081 | 0.009 | 0.062 | 0.70 | 0.015 |
| 9 am to 10 am | 113 (47.1) | 30 (42.9) | 51 (46.8) | 32 (52.5) | 62 (47.3) | 83 (48.8) | 81 (45.3) |  |  |  |  |  |  |  |
| 11 am to 2 pm | 67 (27.9) | 21 (30.0) | 24 (22.0) | 22 (36.1) | 43 (32.8) | 46 (27.1) | 45 (25.1) |  |  |  |  |  |  |  |
| **Clinic visit reasons**^c^ | | | | | | | | | | | | | | |
| PrEP initiation | 24 (10.0) | 12 (17.1) | 12 (11.0) | 0 (0) | 12 (9.2) | 12 (7.1) | 24 (13.4) | 0.004 | 0.24 | <0.001 | 0.007 | 0.63 | 0.018 | 0.003 |
| PrEP follow-up | 86 (35.8) | 58 (82.9) | 28 (25.7) | 0 (0) | 58 (44.3) | 28 (16.5) | 86 (48.0) | <0.001 | <0.001 | <0.001 | <0.001 | 0.003 | <0.001 | <0.001 |
| Outpatient department | 50 (20.8) | 0 (0) | 31 (28.4) | 19 (31.1) | 19 (14.5) | 50 (29.4) | 31 (17.3) | <0.001 | <0.001 | <0.001 | 0.71 | 0.008 | <0.001 | 0.022 |
| HIV testing and counseling | 39 (16.3) | 0 (0) | 25 (22.9) | 14 (23.0) | 14 (10.7) | 39 (22.9) | 25 (14.0) | <0.001 | <0.001 | <0.001 | 1.00 | 0.010 | <0.001 | 0.10 |
| Family planning | 39 (16.3) | 0 (0) | 26 (23.9) | 13 (21.3) | 13 (9.9) | 39 (22.9) | 26 (14.5) | <0.001 | <0.001 | <0.001 | 0.71 | 0.004 | <0.001 | 0.21 |
| Antenatal care | 33 (13.8) | 0 (0) | 20 (18.3) | 13 (21.3) | 13 (9.9) | 33 (19.4) | 20 (11.2) | <0.001 | <0.001 | <0.001 | 0.64 | 0.059 | <0.001 | 0.047 |
| Postnatal care | 3 (1.3) | 0 (0) | 2 (1.8) | 1 (1.6) | 1 (0.8) | 3 (1.8) | 2 (1.1) | 0.53 | 0.25 | 0.28 | 0.93 | 0.46 | 0.26 | 0.75 |
| Child Welfare | 31 (12.9) | 0 (0) | 21 (19.3) | 10 (16.4) | 10 (7.6) | 31 (18.2) | 21 (11.7) | <0.001 | <0.001 | <0.001 | 0.64 | 0.007 | <0.001 | 0.35 |
| Other^d^ | 7 (2.9) | 0 (0) | 5 (4.6) | 2 (3.3) | 2 (1.5) | 7 (4.1) | 5 (2.8) | 0.20 | 0.069 | 0.13 | 0.68 | 0.16 | 0.085 | 0.85 |
| Number of reasons (one) | 180 (75.0) | 70 (100) | 59 (54.1) | 51 (83.6) | 121 (92.4) | 110 (64.7) | 129 (72.1) | <0.001 | <0.001 | 0.002 | <0.001 | <0.001 | <0.001 | 0.16 |
| Two | 49 (20.4) | 0 (0) | 40 (36.7) | 9 (14.8) | 9 (6.9) | 49 (28.8) | 40 (22.3) |  |  |  |  |  |  |  |
| More than two | 11 (4.6) | 0 (0) | 10 (9.2) | 1 (1.6) | 1 (0.8) | 11 (6.5) | 10 (5.6) |  |  |  |  |  |  |  |
| Times survey taken (once) | 220 (91.7) | 57 (81.4) | 103 (94.5) | 60 (98.4) | 117 (89.3) | 163 (95.9) | 160 (89.4) | 0.005 | 0.017 | 0.007 | 0.22 | 0.29 | <0.001 | 0.090 |
| Twice | 19 (7.9) | 12 (17.1) | 6 (5.5) | 1 (1.6) | 13 (9.9) | 7 (4.1) | 18 (10.1) |  |  |  |  |  |  |  |
| More than twice | 1 (0.4) | 1 (1.4) | 0 (0) | 0 (0) | 1 (0.8) | 0 (0) | 1 (0.6) |  |  |  |  |  |  |  |

n (%) or median (interquartile range). ^a^Including three persons who were only counselled about PrEP. ^b^Due to clinic visit. ^c^Multiple reasons possible. ^d^Including two persons accompanying another clinic attendee, two persons collecting antiretroviral therapy for a partner, one person asking to continue PrEP at the clinic, one person attending the clinic for cancer screening, and one person helping at the clinic. Groups were compared using Pearson's χ^2^, Kruskal-Wallis, and Wilcoxon rank-sum tests.

**Table S2:** Out-of-pocket expenses, time spent on clinic visits, and total cost of clinic attendance among PrEP users and other clinic attendees

|  | **Total** | **PrEP only (1)** | **PrEP and other services (2)** | **Other services only (3)** | **(1+3) vs. (2)** | **(1) vs. (2+3)** | **(1+2) vs. (3)** | **P** | **(1) vs. (2)** | **(1) vs. (3)** | **(2) vs. (3)** | **(1+3) vs. (2)** | **(1) vs. (2+3)** | **(1+2) vs. (3)** |
| --- | --- | --- | --- | --- | --- | --- | --- | --- | --- | --- | --- | --- | --- | --- |
|  | **N=240** | **N=70** | **N=109** | **N=61** | **N=131** | **N=170** | **N=179** |  |  |  |  |  |  |  |
| **Out-of-pocket expenses** | | | | | | | | | | | | | | |
| Any OOPE | 190 (79.2) | 46 (65.7) | 92 (84.4) | 52 (85.2) | 98 (74.8) | 144 (84.7) | 138 (77.1) | 0.004 | 0.004 | 0.010 | 0.88 | 0.068 | <0.001 | 0.18 |
| If any, amount ($) | 1.36 (0.91–1.96) | 1.40 (0.91–2.45) | 1.28 (0.91–1.89) | 1.51 (0.94–1.93) | 1.47 (0.91–2.12) | 1.32 (0.91–1.89) | 1.28 (0.91–2.27) | 0.55 | 0.32 | 0.70 | 0.48 | 0.30 | 0.39 | 0.73 |
| **Medical out-of-pocket expenses** | | | | | | | | | | | | | | |
| Any medical OOPE | 76 (31.7) | 5 (7.1) | 44 (40.4) | 27 (44.3) | 32 (24.4) | 71 (41.8) | 49 (27.4) | <0.001 | <0.001 | <0.001 | 0.62 | 0.008 | <0.001 | 0.014 |
| If any, amount ($) | 0.38 (0.38–0.76) | 0.38 (0.38–0.38) | 0.38 (0.38–0.38) | 0.38 (0.38–1.06) | 0.38 (0.38–0.83) | 0.38 (0.38–0.76) | 0.38 (0.38–0.38) | 0.17 | 0.95 | 0.29 | 0.075 | 0.11 | 0.68 | 0.060 |
| Any consultation expenses | 73 (30.4) | 5 (7.1) | 44 (40.4) | 24 (39.3) | 29 (22.1) | 68 (40.0) | 49 (27.4) | <0.001 | <0.001 | <0.001 | 0.90 | 0.002 | <0.001 | 0.079 |
| If any, amount ($) | 0.38 (0.38–0.76) | 0.38 (0.38–0.38) | 0.38 (0.38–0.38) | 0.38 (0.38–1.06) | 0.38 (0.38–0.83) | 0.38 (0.38–0.76) | 0.38 (0.38–0.38) | 0.041 | 0.92 | 0.21 | 0.014 | 0.028 | 0.66 | 0.012 |
| Any medical test expenses | 1 (0.4) | 0 (0) | 0 (0) | 1 (1.6) | 1 (0.8) | 1 (0.6) | 0 (0) | 0.23 |  | 0.28 | 0.18 | 0.36 | 0.52 | 0.086 |
| If any, amount ($) | 0.38 (0.38–0.38) |  |  | 0.38 (0.38–0.38) | 0.38 (0.38–0.38) | 0.38 (0.38–0.38) |  |  |  |  |  |  |  |  |
| Any non-PrEP drug expenses | 7 (2.9) | 0 (0) | 3 (2.8) | 4 (6.6) | 4 (3.1) | 7 (4.1) | 3 (1.7) | 0.083 | 0.16 | 0.030 | 0.23 | 0.89 | 0.085 | 0.050 |
| If any, amount ($) | 0.60 (0.38–0.83) |  | 0.83 (0.60–0.83) | 0.38 (0.26–0.76) | 0.38 (0.26–0.76) | 0.60 (0.38–0.83) | 0.83 (0.60–0.83) | 0.28 |  |  | 0.28 | 0.28 |  | 0.28 |
| Any PrEP drug expenses | 0 (0) | 0 (0) | 0 (0) | 0 (0) | 0 (0) | 0 (0) | 0 (0) |  |  |  |  |  |  |  |
| **Non-medical out-of-pocket expenses** | | | | | | | | | | | | | | |
| Any non-medical OOPE | 176 (73.3) | 46 (65.7) | 84 (77.1) | 46 (75.4) | 92 (70.2) | 130 (76.5) | 130 (72.6) | 0.22 | 0.097 | 0.23 | 0.81 | 0.23 | 0.087 | 0.67 |
| If any, amount ($) | 1.21 (0.91–1.81) | 1.17 (0.91–2.27) | 1.06 (0.91–1.66) | 1.28 (0.91–1.66) | 1.28 (0.91–1.85) | 1.21 (0.91–1.66) | 1.06 (0.91–1.89) | 0.24 | 0.11 | 0.60 | 0.29 | 0.10 | 0.18 | 0.61 |
| Any transport expenses | 158 (65.8) | 44 (62.9) | 74 (67.9) | 40 (65.6) | 84 (64.1) | 114 (67.1) | 118 (65.9) | 0.79 | 0.49 | 0.75 | 0.76 | 0.54 | 0.53 | 0.96 |
| If any, amount (two-way, $) | 1.06 (0.91–1.51) | 1.13 (0.91–2.27) | 1.06 (0.91–1.51) | 1.06 (0.91–1.51) | 1.06 (0.91–1.74) | 1.06 (0.91–1.51) | 1.06 (0.91–1.66) | 0.16 | 0.083 | 0.11 | 0.95 | 0.29 | 0.058 | 0.45 |
| Any food expenses | 56 (23.3) | 9 (12.9) | 26 (23.9) | 21 (34.4) | 30 (22.9) | 47 (27.6) | 35 (19.6) | 0.014 | 0.070 | 0.003 | 0.14 | 0.86 | 0.014 | 0.018 |
| If any, amount ($) | 0.42 (0.30–0.76) | 0.38 (0.38–0.49) | 0.60 (0.30–0.83) | 0.38 (0.30–0.76) | 0.38 (0.30–0.76) | 0.45 (0.30–0.76) | 0.45 (0.30–0.76) | 0.34 | 0.21 | 0.63 | 0.26 | 0.16 | 0.33 | 0.48 |
| Any phone expenses | 38 (15.8) | 8 (11.4) | 18 (16.5) | 12 (19.7) | 20 (15.3) | 30 (17.6) | 26 (14.5) | 0.42 | 0.35 | 0.19 | 0.60 | 0.79 | 0.23 | 0.34 |
| If any, amount ($) | 0.38 (0.15–0.45) | 0.34 (0.15–0.60) | 0.38 (0.20–0.45) | 0.30 (0.15–0.42) | 0.34 (0.15–0.45) | 0.38 (0.15–0.45) | 0.38 (0.18–0.53) | 0.72 | 0.91 | 0.53 | 0.45 | 0.57 | 0.83 | 0.42 |
| Any childcare expenses | 4 (1.7) | 0 (0) | 2 (1.8) | 2 (3.3) | 2 (1.5) | 4 (2.4) | 2 (1.1) | 0.34 | 0.25 | 0.13 | 0.55 | 0.85 | 0.20 | 0.25 |
| If any, amount ($) | 3.78 (2.27–5.67) |  | 5.67 (3.78–7.56) | 2.27 (0.76–3.78) | 2.27 (0.76–3.78) | 3.78 (2.27–5.67) | 5.67 (3.78–7.56) | 0.22 |  |  | 0.22 | 0.22 |  | 0.22 |
| **Other out-of-pocket expenses** | | | | | | | | | | | | | | |
| Any other expenses | 0 (0) | 0 (0) | 0 (0) | 0 (0) | 0 (0) | 0 (0) | 0 (0) |  |  |  |  |  |  |  |
| **Total out-of-pocket expenses** | | | | | | | | | | | | | | |
| Medical OOPE ($) | 0 (0–0.38) | 0 (0–0) | 0 (0–0.38) | 0 (0–0.38) | 0 (0–0) | 0 (0–0.38) | 0 (0–0.38) | <0.001 | <0.001 | <0.001 | 0.34 | 0.022 | <0.001 | 0.005 |
| Non-medical OOPE ($) | 0.91 (0–1.51) | 0.91 (0–1.66) | 0.91 (0.20–1.51) | 1.13 (0.15–1.51) | 0.91 (0–1.59) | 0.91 (0.19–1.51) | 0.91 (0–1.51) | 0.77 | 0.75 | 0.51 | 0.59 | 0.91 | 0.61 | 0.51 |
| All OOPE ($) | 1.06 (0.38–1.81) | 0.91 (0–1.66) | 1.10 (0.57–1.81) | 1.28 (0.76–1.81) | 1.06 (0–1.81) | 1.13 (0.76–1.81) | 1.06 (0.30–1.81) | 0.18 | 0.14 | 0.091 | 0.52 | 0.58 | 0.079 | 0.23 |
| **Time spent on clinic visit** | | | | | | | | | | | | | | |
| Time spent on clinic visits (h) | 3.34 (2.40–4.75) | 3.09 (2.22–4.72) | 3.77 (2.73–4.98) | 2.92 (2.27–3.83) | 3.00 (2.25–4.45) | 3.43 (2.55–4.75) | 3.53 (2.47–4.95) | 0.014 | 0.078 | 0.49 | 0.004 | 0.005 | 0.36 | 0.026 |
| Time spent on clinic visit > 4h | 88 (36.7) | 27 (38.6) | 46 (42.2) | 15 (24.6) | 42 (32.1) | 61 (35.9) | 73 (40.8) | 0.068 | 0.63 | 0.087 | 0.022 | 0.10 | 0.69 | 0.023 |
| Time in clinic (h) | 1.98 (1.15–2.99) | 1.88 (0.90–2.55) | 2.63 (1.47–3.27) | 1.58 (1.00–2.38) | 1.63 (0.90–2.55) | 2.13 (1.25–3.13) | 2.20 (1.20–3.07) | <0.001 | <0.001 | 0.55 | <0.001 | <0.001 | 0.043 | 0.007 |
| PrEP initiation (h) | 2.13 (1.28–3.13) | 2.52 (1.37–2.62) | 2.65 (1.58–3.46) | 1.58 (1.00–2.38) | 1.62 (1.00–2.55) | 2.08 (1.28–3.13) | 2.53 (1.55–3.43) | <0.001 | 0.58 | 0.071 | <0.001 | <0.001 | 0.62 | <0.001 |
| PrEP follow-up (h) | 1.68 (0.90–2.68) | 1.68 (0.90–2.37) | 2.53 (0.82–2.90) | 1.58 (1.00–2.38) | 1.59 (0.90–2.38) | 1.67 (0.90–2.82) | 1.89 (0.88–2.75) | 0.30 | 0.15 | 1.00 | 0.16 | 0.12 | 0.54 | 0.52 |
| Time in clinic > 2h | 119 (49.6) | 30 (42.9) | 69 (63.3) | 20 (32.8) | 50 (38.2) | 89 (52.4) | 99 (55.3) | <0.001 | 0.007 | 0.24 | <0.001 | <0.001 | 0.18 | 0.002 |
| Two-way travel time (h) | 1.00 (0.67–2.00) | 1.17 (0.83–2.00) | 1.00 (0.67–2.00) | 1.00 (0.67–2.00) | 1.17 (0.67–2.00) | 1.00 (0.67–2.00) | 1.00 (0.67–2.00) | 0.40 | 0.18 | 0.39 | 0.71 | 0.29 | 0.19 | 0.87 |
| Two-way travel time > 2h | 34 (14.2) | 13 (18.6) | 13 (11.9) | 8 (13.1) | 21 (16.0) | 21 (12.4) | 26 (14.5) | 0.44 | 0.22 | 0.40 | 0.82 | 0.36 | 0.21 | 0.78 |
| **Opportunity cost of time spent on clinic visit (per minute)** | | | | | | | | | | | | | | |
| Time cost (lost income, ¢/minute) | 0 (0–0) | 0 (0–0) | 0 (0–0) | 0 (0–0) | 0 (0–0) | 0 (0–0) | 0 (0–0) | 0.72 | 0.42 | 0.60 | 0.82 | 0.52 | 0.44 | 0.94 |
| Any time cost (lost income) | 23 (9.6) | 5 (7.1) | 12 (11.0) | 6 (9.8) | 11 (8.4) | 18 (10.6) | 17 (9.5) | 0.69 | 0.39 | 0.58 | 0.81 | 0.49 | 0.41 | 0.94 |
| If any, amount (¢/minute) | 3.03 (0.98–7.27) | 5.56 (3.03–7.27) | 1.79 (1.06–5.29) | 3.48 (0.91–7.41) | 4.91 (0.91–7.41) | 1.98 (0.98–5.38) | 3.03 (1.14–5.56) | 0.61 | 0.29 | 0.58 | 0.85 | 0.46 | 0.33 | 0.94 |
| Time cost (monthly income, ¢/minute) | 1.72 (0.32–8.59) | 5.58 (0.86–13) | 1.03 (0.30–6.01) | 0.52 (0–3.35) | 2.15 (0.34–10) | 0.86 (0–5.15) | 1.72 (0.60–10) | <0.001 | <0.001 | <0.001 | 0.088 | 0.15 | <0.001 | <0.001 |
| Any time cost (monthly income) | 190 (79.2) | 65 (92.9) | 84 (77.1) | 41 (67.2) | 106 (80.9) | 125 (73.5) | 149 (83.2) | 0.001 | 0.006 | <0.001 | 0.16 | 0.46 | <0.001 | 0.008 |
| If any, amount (¢/minute) | 3.39 (0.86–12) | 8.59 (2.15–13) | 1.72 (0.86–9.45) | 1.72 (0.52–6.01) | 4.29 (0.86–13) | 1.72 (0.86–6.87) | 4.29 (0.86–13) | 0.002 | 0.009 | 0.001 | 0.31 | 0.19 | <0.001 | 0.025 |
| Time cost (GDP, ¢/minute) | 0.04 (0.04–0.04) | 0.04 (0.04–0.04) | 0.04 (0.04–0.04) | 0.04 (0.04–0.04) | 0.04 (0.04–0.04) | 0.04 (0.04–0.04) | 0.04 (0.04–0.04) |  |  |  |  |  |  |  |
| **Opportunity cost of time spent on clinic visit (total)** | | | | | | | | | | | | | | |
| Time cost (lost income, $) | 0 (0–0) | 0 (0–0) | 0 (0–0) | 0 (0–0) | 0 (0–0) | 0 (0–0) | 0 (0–0) | 0.68 | 0.38 | 0.57 | 0.81 | 0.49 | 0.40 | 0.93 |
| Any time cost (lost income) | 23 (9.6) | 5 (7.1) | 12 (11.0) | 6 (9.8) | 11 (8.4) | 18 (10.6) | 17 (9.5) | 0.69 | 0.39 | 0.58 | 0.81 | 0.49 | 0.41 | 0.94 |
| If any, amount ($) | 6.00 (3.15–9.58) | 3.15 (3.00–11) | 6.30 (4.18–8.41) | 5.61 (4.33–11) | 4.64 (3.00–11) | 6.29 (4.33–8.52) | 6.00 (3.15–8.52) | 0.92 | 0.71 | 0.72 | 1.00 | 0.83 | 0.68 | 0.89 |
| Time cost (monthly income, $) | 0 (0–0) | 0 (0–0) | 0 (0–0) | 0 (0–0) | 0 (0–0) | 0 (0–0) | 0 (0–0) | 0.83 | 0.93 | 0.57 | 0.60 | 0.81 | 0.75 | 0.54 |
| Any time cost (monthly income) | 41 (17.1) | 13 (18.6) | 19 (17.4) | 9 (14.8) | 22 (16.8) | 28 (16.5) | 32 (17.9) | 0.84 | 0.85 | 0.56 | 0.65 | 0.90 | 0.69 | 0.58 |
| If any, amount ($) | 7.36 (4.71–11) | 6.14 (4.07–10) | 7.67 (6.27–11) | 5.94 (4.85–11) | 6.04 (4.07–11) | 7.50 (5.16–11) | 7.50 (4.71–10) | 0.59 | 0.38 | 0.95 | 0.42 | 0.30 | 0.50 | 0.63 |
| Time cost (GDP, $) | 7.54 (5.42–11) | 6.98 (5.00–11) | 8.50 (6.17–11) | 6.58 (5.12–8.65) | 6.77 (5.08–10) | 7.73 (5.75–11) | 7.97 (5.57–11) | 0.014 | 0.078 | 0.49 | 0.004 | 0.005 | 0.36 | 0.026 |
| **Total cost of clinic attendance** | | | | | | | | | | | | | | |
| Cost of clinic visit (lost income) | 1.21 (0.38–2.27) | 0.91 (0–2.27) | 1.28 (0.76–2.27) | 1.36 (0.91–1.96) | 1.13 (0–2.12) | 1.28 (0.76–2.27) | 1.13 (0.38–2.27) | 0.100 | 0.057 | 0.065 | 0.75 | 0.31 | 0.033 | 0.29 |
| Any cost | 191 (79.6) | 46 (65.7) | 93 (85.3) | 52 (85.2) | 98 (74.8) | 145 (85.3) | 139 (77.7) | 0.003 | 0.002 | 0.010 | 0.99 | 0.044 | <0.001 | 0.20 |
| If any, amount | 1.51 (0.91–2.64) | 1.51 (0.91–2.89) | 1.44 (0.91–2.64) | 1.62 (1.02–2.34) | 1.55 (0.98–2.57) | 1.51 (0.98–2.49) | 1.51 (0.91–2.72) | 0.85 | 0.63 | 0.88 | 0.68 | 0.58 | 0.69 | 0.81 |
| Cost of clinic visit (monthly income) | 1.28 (0.38–2.68) | 1.06 (0–3.14) | 1.28 (0.76–2.72) | 1.51 (0.91–2.34) | 1.28 (0.15–2.64) | 1.32 (0.91–2.64) | 1.21 (0.38–2.95) | 0.38 | 0.21 | 0.25 | 0.83 | 0.50 | 0.17 | 0.50 |
| Any cost | 192 (80.0) | 46 (65.7) | 93 (85.3) | 53 (86.9) | 99 (75.6) | 146 (85.9) | 139 (77.7) | 0.002 | 0.002 | 0.005 | 0.78 | 0.060 | <0.001 | 0.12 |
| If any, amount | 1.66 (1.06–3.83) | 2.27 (1.06–5.62) | 1.51 (0.91–3.32) | 1.66 (1.06–2.57) | 1.81 (1.06–4.27) | 1.62 (0.98–2.95) | 1.66 (1.06–4.53) | 0.28 | 0.15 | 0.15 | 0.96 | 0.38 | 0.11 | 0.58 |
| Cost of clinic visit (GDP) | 8.92 (6.51–12) | 8.34 (6.21–12) | 10.17 (7.15–13) | 8.02 (6.36–11) | 8.13 (6.21–12) | 9.11 (6.62–12) | 9.38 (6.51–13) | 0.054 | 0.14 | 0.62 | 0.016 | 0.021 | 0.43 | 0.070 |
| Any cost | 240 (100) | 70 (100) | 109 (100) | 61 (100) | 131 (100) | 170 (100) | 179 (100) |  |  |  |  |  |  |  |
| If any, amount | 8.92 (6.51–12) | 8.34 (6.21–12) | 10 (7.15–13) | 8.02 (6.36–11) | 8.13 (6.21–12) | 9.11 (6.62–12) | 9.38 (6.51–13) | 0.054 | 0.14 | 0.62 | 0.016 | 0.021 | 0.43 | 0.070 |

n (%) or median (interquartile range). GDP = gross domestic product. OOPE = Out-of-pocket expenses. ^a^Time valued with the median of ¢3.023 per minute lost income of those who reported lost income. ^b^ Everyone’s time valued with a per-capita GDP of ¢3.761 per minute worktime. Groups were compared using Pearson's χ^2^, Kruskal-Wallis, and Wilcoxon rank-sum tests.

**Table S3:** Regression analyses of the relationships of out-of-pocket expenses, time spent on clinic visit, and total cost of clinic attendance with clinic visit reasons

| **Covariates (model)** | **(1.1) Any medical OOPE (yes/no)^a^** ***OR*** | **(2.1) Any non-medical OOPE (yes/no)^a^ *OR*** | **(3) Time in clinic (minutes) *Coef.*** | **(5) Time spent on clinic visit (minutes) *Coef.*** | **(7.1) Any costs of clinic attendance (yes/no)^a,c^ *OR*** |
| --- | --- | --- | --- | --- | --- |
| **Clinic visit reason** | | | | | |
| PrEP initiation | 1.01 (0.52–1.96) | 1.19 (0.55–2.59) | 53 (30–76)^***^ | 50 (18–81)^**^ | 1.22 (0.47–3.17) |
| PrEP follow-up | 0.52 (0.22–1.22) | 0.89 (0.37–2.14) | 21 (−8.88–50) | 20 (−17–58) | 0.65 (0.24–1.80) |
| Other | 6.26 (2.34–17)^***^ | 1.50 (0.72–3.12) | 18 (−9.19–45) | 3.23 (−35–41) | 2.19 (0.99–4.85) |
| Constant | 0.13 (0.042–0.38)^***^ | 2.05 (0.80–5.23) | 87 (56–119)^***^ | 189 (145–233)^***^ | 2.64 (0.91–7.67) |
| Pseudo or adjusted R^2^ | 0.12 | 0.012 | 0.085 | 0.026 | 0.053 |
| **Covariates (model)** | **(1.2) If any medical OOPE, amount ($)^a^ *Coef.*** | **(2.2) If any non-medical OOPE, amount ($)^a^ *Coef.*** | **(4) Two-way travel time (minutes) *Coef.*** | **(6) Cost of clinic attendance ($)^b^ *Coef.*** | **(7.2) If any costs of clinic attendance, amount ($)^a,c^ *Coef.*** |
| **Clinic visit reason** | | | | | |
| PrEP initiation | −0.20 (−0.53–0.12) | 0.10 (−0.21–0.40) | −3.34 (−26–19) | 1.98 (0.62–3.35)^**^ | 0.083 (−0.31–0.48) |
| PrEP follow-up | −0.35 (−0.91–0.21) | −0.039 (−0.34–0.26) | −0.15 (−27–27) | 0.50 (−1.03–2.04) | −0.17 (−0.65–0.31) |
| Other | 0.024 (−0.56–0.61) | −0.26 (−0.57–0.053) | −14 (−41–12) | 0.050 (−1.48–1.58) | −0.17 (−0.59–0.25) |
| Constant | −0.44 (−1.07–0.20) | 0.65 (0.29–1.01)^***^ | 102 (71–133)^***^ | 8.60 (6.79–10)^***^ | 1.07 (0.55–1.59)^***^ |
| AIC/BIC or adjusted R^2^ | 0.96/−289 | 3.02/−807 | −0.0023 | 0.025 | 3.89/−828 |

^*^P < 0.05, ^**^P < 0.01, ^***^P < 0.001. ^a^Two-part regression model combining a logit model and a linear regression model. ^b^Based on time valuation with per-capita gross domestic product. ^c^Based on time valuation with median forgone earnings for those who reported forgone earnings. ^d^Clinic visit reasons were included as binary variables (yes/no) in the regression models; (no) was the reference in logit models. Other reason includes outpatient department, HIV testing and counseling, family planning, ante- and postnatal care, and child welfare among other services.

**Table S4:** Regression analyses of the relationships of out-of-pocket expenses, time spent on clinic visit, and total cost of clinic attendance with clinic visit reasons

| **Covariates (model)** | **(1.1) Any medical OOPE (yes/no)^a^** ***OR (95% CI)*** | **(2.1) Any non-medical OOPE (yes/no)^a^ *OR (95% CI)*** | **(3) Time in clinic (minutes) *Coef. (95% CI)*** | **(5) Time spent on clinic visit (minutes) *Coef. (95% CI)*** | **(7.1) Any costs of clinic attendance (yes/no)^a,c^ *OR (95% CI)*** |
| --- | --- | --- | --- | --- | --- |
| **Clinic visit reason** | | | | | |
| PrEP initiation | 0.99 (0.42–2.34) | 1.24 (0.51–2.99) | 55.0 (31.6–78.4)^***^ | 54.1 (21.6–86.6)^**^ | 1.17 (0.40–3.45) |
| PrEP follow-up | 0.60 (0.22–1.62) | 0.70 (0.29–1.66) | 20.5 (−4.70–45.7) | 25.6 (−7.24–58.5) | 0.60 (0.21–1.72) |
| Outpatient department | 33.0 (11.3–96.3)^***^ | 0.93 (0.40–2.17) | −0.19 (−27.1–26.7) | −16.6 (−52.2–19.0) | 2.89 (0.87–9.67) |
| HIV testing and counseling | 0.93 (0.33–2.64) | 0.60 (0.27–1.34) | 6.62 (−18.5–31.7) | 14.5 (−20.1–49.1) | 0.76 (0.3–1.92) |
| Family planning | 2.47 (0.87–7.01) | 1.24 (0.49–3.13) | 6.48 (−19.4–32.3) | −7.52 (−41.7–26.7) | 1.87 (0.65–5.34) |
| Ante- or postnatal care | 6.02 (2.08–17.4)^***^ | 1.59 (0.6–4.16) | 57.1 (28.0–86.3)^***^ | 64.0 (27.4–101)^***^ | 2.82 (0.88–9.07) |
| Child Welfare | 2.84 (0.91–8.81) | 2.22 (0.79–6.23) | 14.6 (−10.5–39.6) | 2.01 (−31.2–35.2) | 1.77 (0.55–5.71) |
| Other | 0.52 (0.12–2.31) | 2.46 (0.26–23.1) | 24.8 (−25.2–74.8) | 42.5 (−37.6–122) | 1.95 (0.21–18.2) |
| **PrEP promotion** | | | | | |
| PrEP promotion package | 0.91 (0.30–2.75) | 0.83 (0.30–2.27) | −0.37 (−33.9–33.2) | −2.62 (−46.0–40.8) | 0.99 (0.29–3.41) |
| Constant | 0.13 (0.04–0.35)^***^ | 2.70 (1.14–6.42)^*^ | 86.0 (60.9–111)^***^ | 179 (146–214)^***^ | 2.97 (1.02–8.62)^*^ |
| Pseudo or adjusted R^2^ | 0.29 | 0.031 | 0.12 | 0.066 | 0.069 |
| **Covariates (model)** | **(1.2) If any medical OOPE, amount ($)^a^ *Coef. (95% CI)*** | **(2.2) If any non-medical OOPE, amount ($)^a^ *Coef. (95% CI)*** | **(4) Two-way travel time (minutes) *Coef. (95% CI)*** | **(6) Cost of clinic attendance ($)^b^ *Coef. (95% CI)*** | **(7.2) If any costs of clinic attendance, amount ($)^a,c^ *Coef. (95% CI)*** |
| **Reason for clinic visit** | | | | | |
| PrEP initiation | −0.027 (−0.42–0.36) | 0.11 (−0.19–0.42) | −0.89 (−25.7–23.9) | 2.31 (0.80–3.82)^**^ | −0.0087 (−0.41–0.39) |
| PrEP follow-up | −0.17 (−0.66–0.32) | 0.017 (−0.26–0.30) | 5.14 (−19.3–29.6) | 0.81 (−0.62–2.23) | −0.12 (−0.54–0.31) |
| Outpatient department | 0.41 (0.032–0.79)^*^ | −0.18 (−0.48–0.11) | −16.4 (−42.1–9.28) | −0.40 (−1.98–1.19) | −0.066 (−0.45–0.32) |
| HIV testing and counseling | −0.29 (−0.62–0.047) | 0.30 (−0.06–0.66) | 7.89 (−19.8–35.6) | 0.76 (−0.86–2.38) | 0.31 (−0.10–0.72) |
| Family planning | −0.096 (−0.55–0.36) | −0.12 (−0.44–0.20) | −14.0 (−41.7–13.7) | −0.37 (−1.92–1.17) | −0.28 (−0.63–0.06) |
| Ante- or postnatal care | 0.048 (−0.42–0.52) | −0.31 (−0.62–0.0046) | 6.83 (−22.3–36.0) | 2.28 (0.72–3.84)^**^ | −0.27 (−0.75–0.22) |
| Child Welfare | 0.27 (−0.20–0.74) | −0.30 (−0.60–−0.0046)^*^ | −12.6 (−38.4–13.3) | −0.050 (−1.51–1.41) | 0.0016 (−0.44–0.44) |
| Other | 0.67 (0.34–1.01)^***^ | −0.16 (−0.74–0.41) | 17.6 (−29.6–64.9) | 1.64 (−1.91–5.19) | −0.47 (−1.10–0.17) |
| **PrEP promotion** | | | | | |
| PrEP promotion package | −0.31 (−0.71–0.10) | −0.17 (−0.57–0.23) | −2.25 (−31.1–26.6) | −0.50 (−2.37–1.37) | 0.23 (−0.27–0.73) |
| Constant | −0.72 (−1.21–−0.22)^**^ | 0.56 (0.27–0.85)^***^ | 93.7 (68.0–119)^***^ | 8.11 (6.60–9.63)^***^ | 0.99 (0.57–1.41)^***^ |
| AIC/BIC or adjusted R^2^ | 1.04/−272 | 3.06/−781 | −0.012 | 0.047 | 3.92/−803 |

^*^P < 0.05, ^**^P < 0.01, ^***^P < 0.001. ^a^Two-part regression model combining a logit model and a linear regression model. ^b^Based on time valuation with per-capita gross domestic product. ^c^Based on time valuation with median forgone earnings for those who reported forgone earnings. ^d^Clinic visit reasons and PrEP promotion were included as binary variables (yes/no) in the regression models; (no) was the reference in logit models.

**Table S5:** Regression analyses of the relationships of out-of-pocket expenses, time spent on clinic visit, and total cost of clinic attendance with clinic visit reasons

| **Covariates (model)** | **(1.1) Any medical OOPE (yes/no)^a^** ***OR*** | **(2.1) Any non-medical OOPE (yes/no)^a^ *OR*** | **(3) Time in clinic (minutes) *Coef.*** | **(5) Time spent on clinic visit (minutes) *Coef.*** | **(7.1) Any costs of clinic attendance (yes/no)^a,c^ *OR*** |
| --- | --- | --- | --- | --- | --- |
| **Clinic visit reason** | | | | | |
| PrEP initiation | 0.86 (0.41–1.80) | 1.30 (0.54–3.12) | 54 (31–78)^***^ | 53 (19–88)^**^ | 1.18 (0.41–3.40) |
| PrEP follow-up | 0.53 (0.23–1.24) | 0.87 (0.37–2.10) | 20 (−9.11–50) | 20 (−18–57) | 0.66 (0.24–1.81) |
| Other | 6.58 (2.43–18)^***^ | 1.46 (0.71–3.04) | 17 (−9.40–44) | 2.20 (−36–40) | 2.21 (1–4.89) |
| **Control variable** | | | | | |
| PrEP promotion package | 1.55 (0.65–3.69) | 0.80 (0.29–2.18) | −3.63 (−38–31) | −10 (−55–35) | 1.10 (0.32–3.72) |
| Constant | 0.12 (0.039–0.37)^***^ | 2.09 (0.82–5.33) | 88 (56–120)^***^ | 190 (146–234)^***^ | 2.62 (0.90–7.59) |
| Pseudo or adjusted R^2^ | 0.12 | 0.013 | 0.081 | 0.022 | 0.053 |
| **Covariates (model)** | **(1.2) If any medical OOPE, amount ($)^a^ *Coef.*** | **(2.2) If any non-medical OOPE, amount ($)^a^ *Coef.*** | **(4) Two-way travel time (minutes) *Coef.*** | **(6) Cost of clinic attendance ($)^b^ *Coef.*** | **(7.2) If any costs of clinic attendance, amount ($)^a,c^ *Coef.*** |
| **Clinic visit reason** | | | | | |
| PrEP initiation | −0.10 (−0.48–0.28) | 0.19 (−0.18–0.55) | −0.99 (−27–25) | 2.26 (0.69–3.83)^**^ | 0.039 (−0.39–0.47) |
| PrEP follow-up | −0.35 (−0.91–0.21) | −0.074 (−0.37–0.22) | −0.59 (−28–27) | 0.45 (−1.08–1.98) | −0.15 (−0.64–0.33) |
| Other | 0.023 (−0.56–0.61) | −0.32 (−0.63–−0.0097)^*^ | −15 (−41–11) | −0.029 (−1.56–1.50) | −0.15 (−0.58–0.28) |
| **Control variable** | | | | | |
| PrEP promotion package | −0.28 (−0.65–0.093) | −0.31 (−0.71–0.099) | −6.48 (−35–22) | −0.77 (−2.69–1.14) | 0.12 (−0.38–0.63) |
| Constant | −0.44 (−1.07–0.20) | 0.71 (0.35–1.07)^***^ | 103 (71–134)^***^ | 8.68 (6.87–10)^***^ | 1.04 (0.51–1.57)^***^ |
| AIC/BIC or adjusted R^2^ | 0.98/−286 | 3.02/−803 | −0.0058 | 0.024 | 3.89/−823 |

^*^P < 0.05, ^**^P < 0.01, ^***^P < 0.001. ^a^Two-part regression model combining a logit model and a linear regression model. ^b^Based on time valuation with per-capita gross domestic product. ^c^Based on time valuation with median forgone earnings for those who reported forgone earnings. ^d^Clinic visit reasons and PrEP promotion were included as binary variables (yes/no) in the regression models; (no) was the reference in logit models. Other reason includes outpatient department, HIV testing and counseling, family planning, ante- and postnatal care, and child welfare among other services.
